# Ligand design for Rh(iii)-catalyzed C–H activation: an unsymmetrical cyclopentadienyl group enables a regioselective synthesis of dihydroisoquinolones†
†Electronic supplementary information (ESI) available: Experimental procedures and compound characterization. CCDC 1021286. For ESI and crystallographic data in CIF or other electronic format see DOI: 10.1039/c4sc02590c



**DOI:** 10.1039/c4sc02590c

**Published:** 2014-10-01

**Authors:** Todd K. Hyster, Derek M. Dalton, Tomislav Rovis

**Affiliations:** a Department of Chemistry , Colorado State University , Fort Collins , Colorado 80523 , USA . Email: rovis@lamar.colostate.edu

## Abstract

We report the regioselective synthesis of dihydroisoquinolones from aliphatic alkenes and *O*-pivaloyl benzhydroxamic acids mediated by a Rh(iii) precatalyst bearing sterically bulky substituents. While the prototypical Cp* ligand provides product with low selectivity, sterically bulky Cp^*t*^ affords product with excellent regioselectivity for a range of benzhydroxamic acids and alkenes. Crystallographic evidence offers insight as to the source of the increased regioselectivity.

## Abstract 2

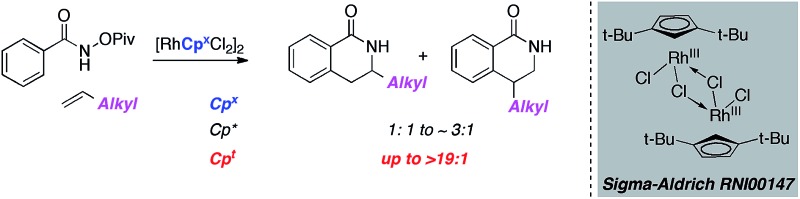
A modified cyclopentadienyl ligand greatly improves regioselectivity in Rh(iii) catalyzed alkene insertion/C–H activation.

C–H activation mediated processes have provided a unique retrosynthetic approach to access a variety of substituted heterocycles.^1^ One tactic that has received increased attention is the coupling of π-components with heteroatom containing molecules.^2^ A variety of transition metals are capable of catalyzing this type of transformation, providing access to dozens of heterocyclic motifs.^1–3^ A challenge for these methods is controlling the regioselectivity of migratory insertion across alkenes and alkynes after the metallacycle forming C–H activation (eqn 1).

Steric and electronic effects are understood to control migratory insertion of unsymmetrical alkynes in Rh(iii) catalyzed isoquinolone syntheses (eqn 1). When the substituents are electronically similar, the larger group resides β- to Rh in the metallacycle to avoid unfavorable steric interactions (selectivity is generally >10 : 1).^4^ When the substituents are electronically different, the more electron-donating group prefers being α- to rhodium in the metallacycle, presumably to stabilize the electron poor metal.^5,6^ The type of C–H bond being activated also plays an important role in the regioselectivity of migratory insertion; aromatic substrates typically provide synthetically useful regioselectivities when electronically different alkynes are used (>10 : 1) but alkenyl C–H activation leads to products with lower regioselectivities, presumably due to minimal steric interactions during migratory insertion.^7,8^ We found that sterically bulky di-*tert*-butylcyclopentadienyl ligand (Cp^*t*^) enhances the regioselectivity of the alkyne migratory insertion event in these cases, delivering regioselectivities (>10 : 1) modestly above those achievable by Cp* ligated Rh complexes (<6 : 1). However, when the alkyne migratory insertion was poorly selective with RhCp* (<3 : 1), RhCp^*t*^ complex was ineffective at providing synthetically useful levels of selectivity. Furthermore, the Cp^*t*^ ligand was only effective with aryl substituted alkynes, presumably because of strong steric interactions between the ligand and alkyne in the insertion event.
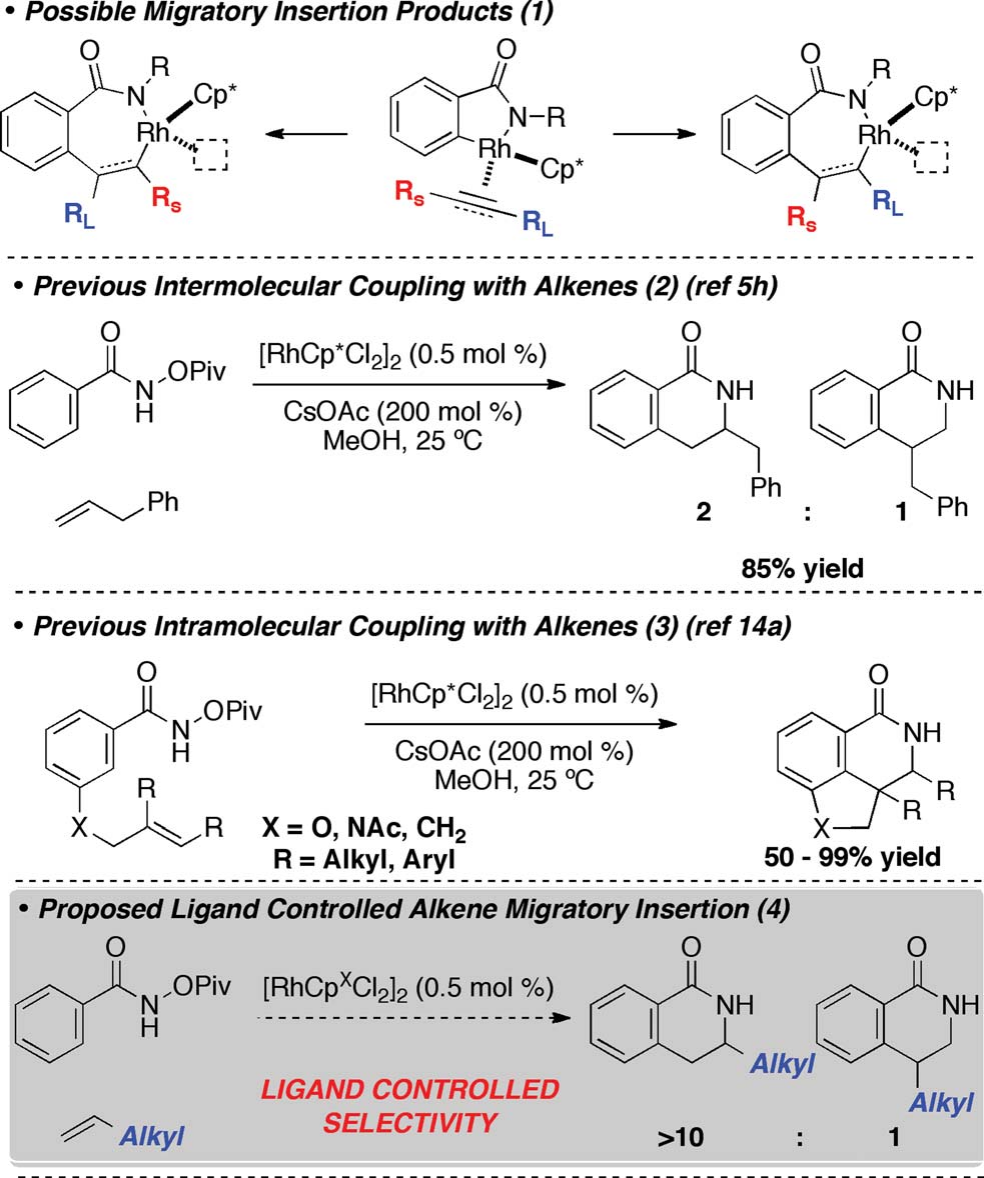


Migratory insertion of alkenes to access heterocycles using C–H activation chemistry is still relatively rare, with seminal studies by Glorius and Fagnou reporting the synthesis of dihydroisoquinolones.^9–11^ Similar to alkynes, alkenyl electron-donating groups favor the position adjacent to the metal in the metallacycle delivering high regioselectivity. In contrast to alkynes, aliphatic alkenes afford product with poor regioselectivity (2 : 1) (eqn 2).^5h,12^ We hypothesized competing steric and electronic effects cause the low regioselectivity, with steric effects favoring the formation of a 4-substituted product and electronics favoring the formation of a 3-substituted product.^13^ As a temporary solution to this problem, our group and others have employed tethering strategies to increase the regioselectivity of the migratory insertion event (eqn 3).^14,15^ Of course, regioselectivity controlled by the ligand on Rh would be the optimal solution to the selectivity problem (eqn 4).^16^ Consequently, we focused our attention toward developing an intermolecular variant of this reaction that would provide product with improved regioselectivity.

As a model system, we explored the impact ligands have on the coupling of *O*-pivaloyl-benzhydroxamic acid **1a** with 1-decene **2a** to provide dihydroisoquinolones **3a** and **3a′**. When Cp* is used as a ligand, the desired products are isolated in excellent yield but poor selectivity (2.4 : 1 **3a** : **3a′**) (Table 1, entry 1).

**Table 1:** Ligand optimization^*a*^

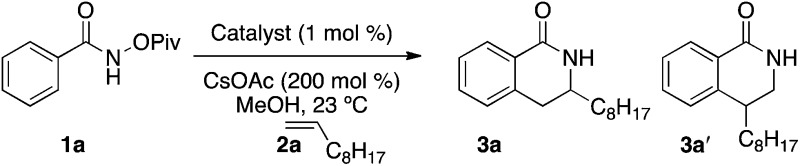			
Entry	Catalyst	Yield (%)	Regioselectivity
1	[RhCp*Cl_2_]_2_	90	2.4 : 1
2^*b*^	[RhCp^CF3^Cl_2_]_2_	85	2.4 : 1
3^*c*^	[RhCp^‡^Cl_2_]_2_	82	12 : 1
4^*d*^	[RhCp^*t*^Cl_2_]_2_	92	15 : 1

^*a*^Reaction conditions: **1a** (.2 mmol), 1-decene (.2 mmol), precatalyst (1 mol%), CsOAc (200 mol%), MeOH (0.1 M).

^*b*^Cp^CF3^ = 1-trifluoromethyl-2-3,4,5-tetramethylcyclopentadienyl.

^*c*^Cp^‡^ = 1,2-di-phenyl-3,4,5-trimethylcyclopentadienyl.

^*d*^Cp^*t*^ = 1,3-di-*t*-butylcyclopentadienyl.

To determine the effect that ligand electronics have on product regioselectivity, we employed an electron deficient 1-trifluoromethyl-2,3,4,5-tetramethylcyclopentadienyl ligand originally developed by Gassman (Cp^CF3^)^17^ and found that this catalyst provides **3a** and **3a′** products in good yield but without an increase in selectivity (2.4 : 1) (Table 1, entry 2).^18,19^ Since ligand electronics did not appear to affect product regioselectivity, we tested an electron rich, sterically bulky di-phenyl-tri-methyl Cp ligand (Cp^‡^) and were pleased to find a remarkable increase in selectivity from 2.4 : 1 to 12 : 1 (**3a** : **3a′**). Pleased by this improvement, we tested the sterically bulky di-*tert*-butyl Cp ligand Cp^*t*^ and were surprised to find that RhCp^*t*^ provides the desired product in 91% yield with exquisite regioselectivity (15 : 1) (Table 1, entry 4).

Having demonstrated that Cp^*t*^ is able to substantially increase the regioselectivity of alkene migratory insertion, we explored the scope of *O*-pivaloyl benzyhydroxamic acids amenable to this reaction. We were pleased to find *para*-substituted benzhydroxamic acids are well tolerated (50–76% yields) with both Cp* and Cp^*t*^ ligands. Notably, the low regioselectivity of <2.2 : 1 seen using [RhCp*Cl_2_]_2_ is dramatically improved with [RhCp^*t*^Cl_2_]_2_ giving excellent regioselectivities of >14 : 1 (Table 2, entries 1–4). Electron rich amides derived from gallic acid provide product in excellent yield and regioselectivity (Table 2, entry 5). Finally, heterocyclic amides are both well tolerated under the reaction conditions and responsive to the sterically bulky ligand, providing product with excellent regioselectivity (Table 2, entries 6–7).

**Table 2:** Amide scope^*a*^

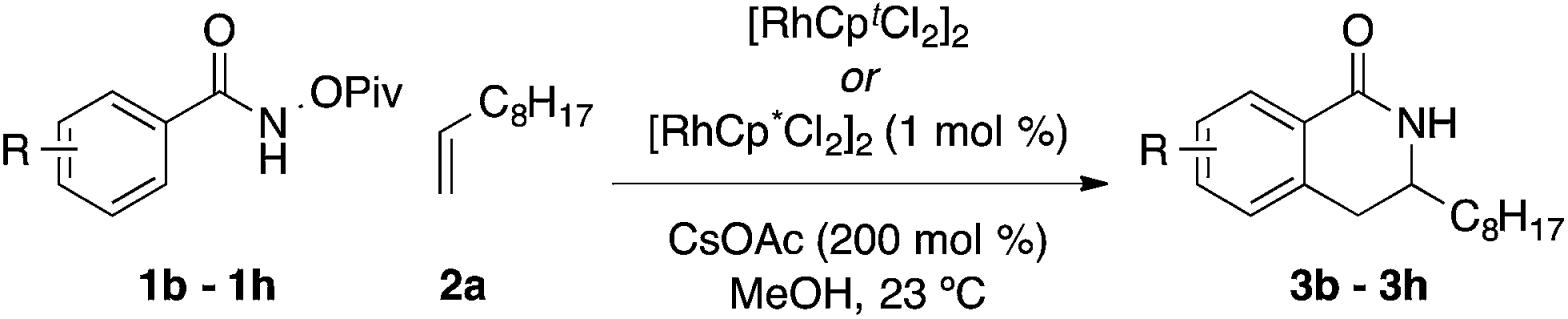				
Entry	Starting material	Yield^*b*^ (%)	Cp*	Cp^*t*^
	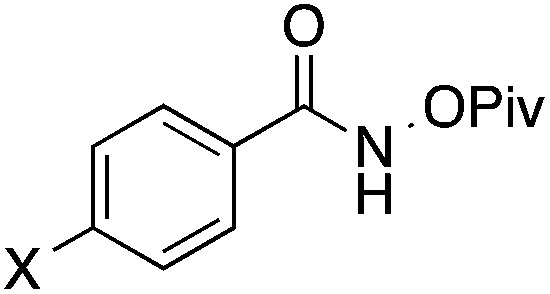			
1	X = CF_3_ (**1b**)	50	1.5 : 1	19 : 1
2	X = Cl (**1c**)	76	2.2 : 1	19 : 1
3	X = OMe (**1d**)	70	1.9 : 1	16 : 1
4	X = Ph (**1e**)	75	1.7 : 1	14 : 1
5	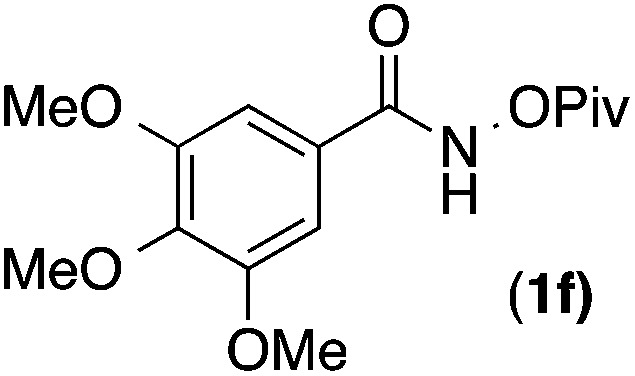	95	1.9 : 1	15 : 1
6	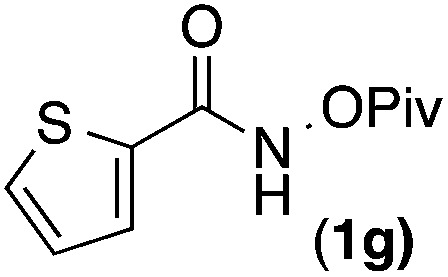	84	2.5 : 1	19 : 1
7	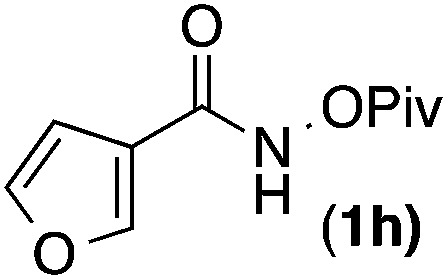	88	1.8 : 1	19 : 1

^*a*^Reaction conditions: amide (.2 mmol), 1-decene (.2 mmol), precatalyst (1 mol%), CsOAc (200 mol%), MeOH (0.1 M).

^*b*^Isolated yield of reaction using [RhCp^*t*^Cl_2_]_2_ as a precatalyst.

*meta*-Substituents also provide exquisite levels of regioselectivity for alkene migratory insertion when Cp^*t*^ is used (>15 : 1) (Table 3). Interestingly, when Cp* is used the C–H activation occurs exclusively at the 6-position; we suggest that this selectivity is the result of steric interactions between the 2-substituent and metal complex during concerted metallation deprotonation. Surprisingly, when Cp^*t*^ is used, the regioselectivity of the C–H activation actually decreases to afford a mixture of 2- and 6-substituted products. While on the surface the decrease of selectivity appears counterintuitive, it can be explained by the uneven distribution of steric bulk in the Cp^*t*^ ligand, which we will comment on subsequently. Not only is this decrease in selectivity mechanistically intriguing, it also offers an exciting opportunity to potentially reverse the regioselectivity of C–H activation.

**Table 3:** Influence of Cp^*t*^ on C–H insertion selectivity^*a*^

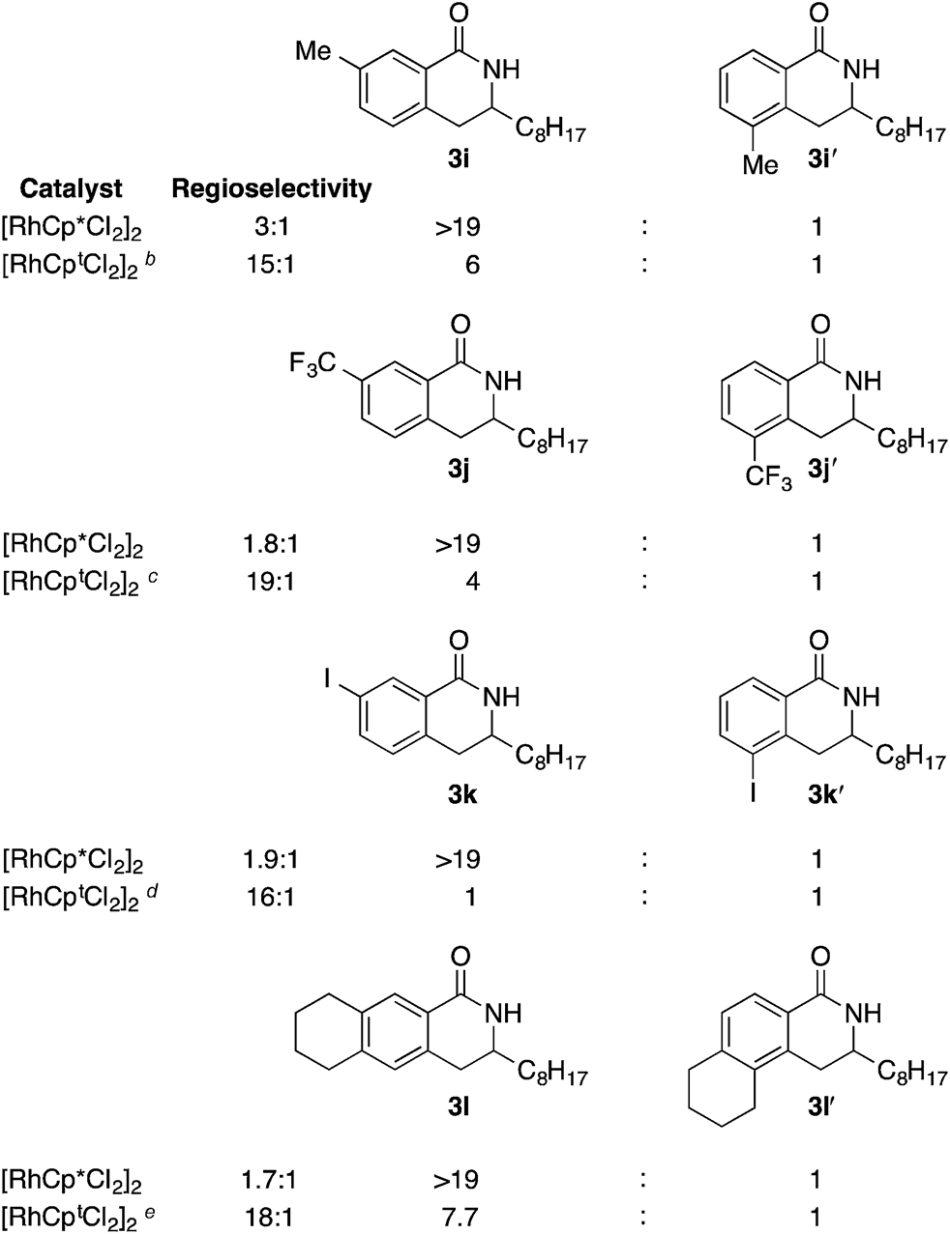

^*a*^Reaction conditions: amide (.2 mmol), 1-decene (.2 mmol), precatalyst (1 mol%), CsOAc (200 mol%), MeOH (0.1 M). isolated yield of reaction using [RhCp^*t*^Cl_2_]_2_ as a precatalyst.

^*b*^67% yield.

^*c*^80% yield.

^*d*^85% yield.

^*e*^79% yield.

We next explored the alkene tolerance of the method. Allyl benzene **2b** furnishes a 1.6 : 1 ratio of dihydroisoquinolone with RhCp* (Table 4, entry 1). When Cp^*t*^ is employed, the regioselectivity increases to 4.5 : 1. Reducing the temperature to 0 °C further increases regioselectivity to 5.1 : 1. Placing an electron-donating group on the aromatic ring increases the selectivity of insertion (Table 4, entry 2). Aryl electron withdrawing substituents do not, however, provide a change in regioselectivity relative to phenyl (Table 4, entry 3). When the aromatic ring is moved further from the alkene the regioselectivity observed with Cp^*t*^ increases and yields remain high (Table 4, entries 4 and 5). Ester and ketone functional groups are tolerated without a detrimental impact on regioselectivity (Table 4, entry 6, 9, and 10). Unfortunately, alkenes bearing stereocenters are ineffective at inducing diastereoselectivity with either ligand (Table 4, entries 6 and 8). Vinyl cyclopropanes participate with good levels of regioselectivity but poor diastereoselectivity (Table 4, entry 8). Unprotected alcohols provide product in excellent yield with no sign of oxidation (Table 4, entry 7).

**Table 4:** Impact of Cp^*t*^ on alkene scope^*a*^

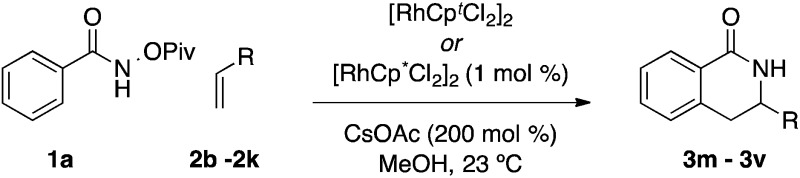				
Entry	Alkene	Yield^*b*^ (%)	Cp*	Cp^*t*^
1^*c*^	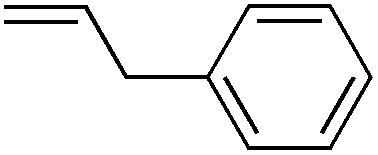	85	1.6 : 1	5.1 : 1
2	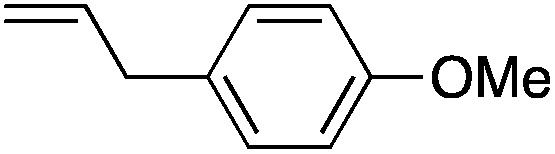	68	1.6 : 1	9.4 : 1
3	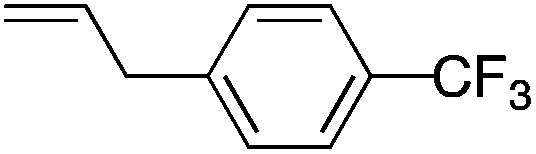	70	1.3 : 1	5.5 : 1
4	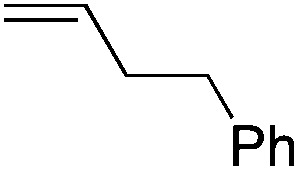	95	2.3 : 1	14 : 1
5	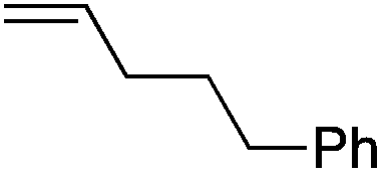	85	1.6 : 1	8 : 1
6^*d*^	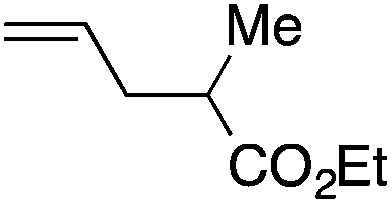	92	1.2 : 1	7.2 : 1
7	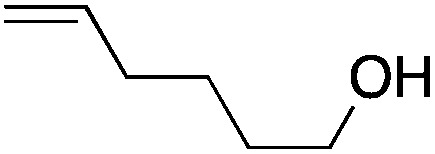	80	1.4 : 1	12 : 1
8^*e*^	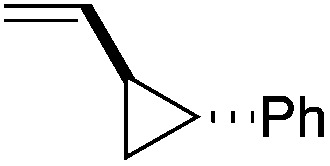	93	1 : 1	11 : 1
9	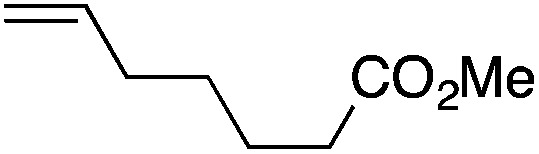	89	2 : 1	14 : 1
10	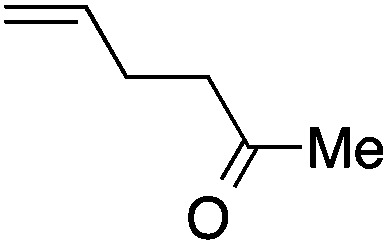	94	3 : 1	14 : 1

^*a*^Reaction conditions: **1a** (.2 mmol), alkene (.2 mmol), precatalyst (1 mol%), CsOAc (200 mol%), MeOH (0.1 M).

^*b*^Isolated yield of reaction using [RhCp^*t*^Cl_2_]_2_ as a precatalyst.

^*c*^Reaction conducted at 0 °C.

^*d*^Products isolated as a 1 : 1 ratio of diastereomers.

^*e*^Product isolated as a 2 : 1 ratio of diastereomers.

While it is desirable to achieve high regioselectivity for a single regioisomer, it is even more attractive to use a ligand to access alternate regioisomers. Currently, the only example of Rh(iii)-catalyzed synthesis of 4-substituted dihydroisoquinolones is with potassium vinyltrifluoroborates where electronics are believed to control regioselectivity.^20^ We found that when vinylcyclohexane was submitted to a reaction with [RhCp*Cl_2_]_2_ as the precatalyst, the 3-substituted dihydroisoquinolone **4a** was isolated in 90% yield with 11 : 1 regioselectivity (Fig. 1). However, when the same reaction was catalyzed by [RhCp^*t*^Cl_2_]_2_ the opposite isomer **4b** was isolated in 75% yield and 10 : 1 (**4b** : **4a**) regioselectivity. Given this unexpected discovery, we were interested in gleaning insight into how Cp^*t*^ influences regioselectivity of alkene migratory insertion. A competition experiment between vinyl cyclohexane **2m** and 1-decene **2a** run to 10% conversion favored the formation of dihydroisoquinolone **3a** in >19 : 1 ratio as determined by ^1^H NMR. This experiment suggests that enhanced steric interactions between the substrate and ligand slow the rate of migratory insertion.

**Fig. 1:**
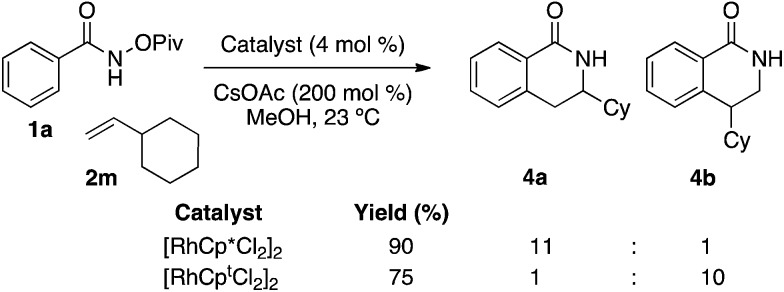
Impact of ligand on reaction of vinyl cyclohexane.

To investigate the steric differences between the RhCp* and RhCp^*t*^ systems X-ray analysis was conducted on a 5-membered RhCp^*t*^ metallacycle. While we were unable to obtain a 5-membered rhodacycle from our system, Jones and coworkers previously characterized 5-membered rhodacycle **5a** from *N*-benzylidenemethanamine and [RhCp*Cl_2_]_2_.^21^ We found that a similar metallacycle **5b** derived from [RhCp^*t*^Cl_2_]_2_ could be obtained in crystalline form under identical conditions and was evaluated by single crystal X-ray diffraction.

A comparison of the bond lengths and angles reveals several notable differences between our Cp^*t*^ rhodacycle and the Cp* rhodacycle reported by Jones (Fig. 2). The Rh–Cp centroid distance in **5b** is 0.011 Å longer than **5a** which is either the result of increased steric interactions, or an artifact of Cp^*t*^ being a less electron-donating ligand. While there are subtle differences in many bond lengths and angles, the most striking difference is the angle C3–Rh–Cl, which is 98.03° in **5b** while only 90.09° in **5a**. The angle increase is likely the result of steric interactions caused by the *tert*-butyl moiety being situated directly over the Rh–Cl bond. As alkene exchange presumably occurs with Cl, we suggest that steric interactions between the *t*-butyl of the ligand and the alkene substituent affect both the alkene coordination and 1,2-insertion events.

**Fig. 2:**
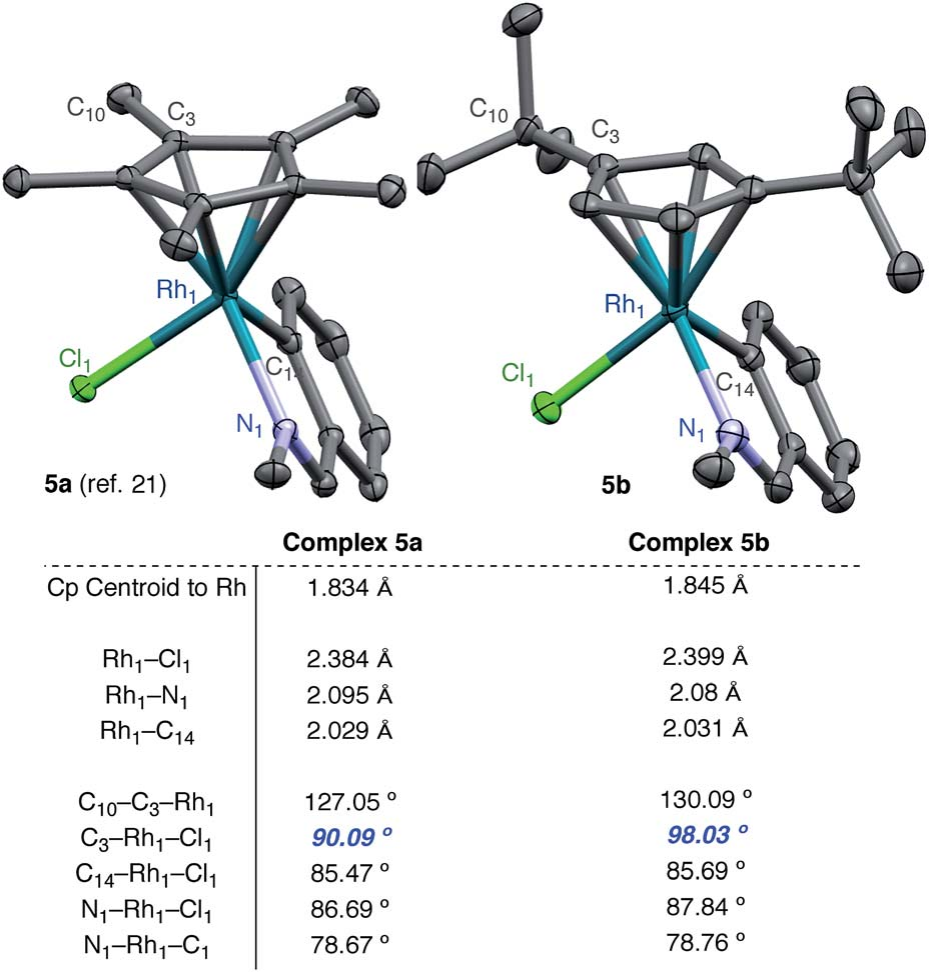
X-Ray analysis.

Based on the X-ray crystal structure and regioselectivity data, we propose the following model for regioselectivity of the 1,2-migratory insertion of alkenes, where steric contributions from the *t*-butyl groups influence both alkene coordination and insertion events to give high selectivity. With small alkyl alkenes, we propose that steric interactions from one *t*-butyl of Cp^*t*^ disfavor alkene coordination (**I**) and subsequent insertion to give the β-substituted product **3a′** (Fig. 3). Coordination of the alkene with the steric bulk oriented away from the *t*-butyl group finds minimized steric interactions during coordination (**II**). Subsequent migratory insertion from **II** places the alkyl substituent α to Rh in the transition state, which we propose is able to stabilize a buildup of partial positive charge, making the α-substituted product **3a** both sterically and electronically favored with Cp^*t*^. In the case of the Cp* ligand with small alkyl alkenes, neither steric nor electronic interactions dominate so low selectivity is observed.

**Fig. 3:**
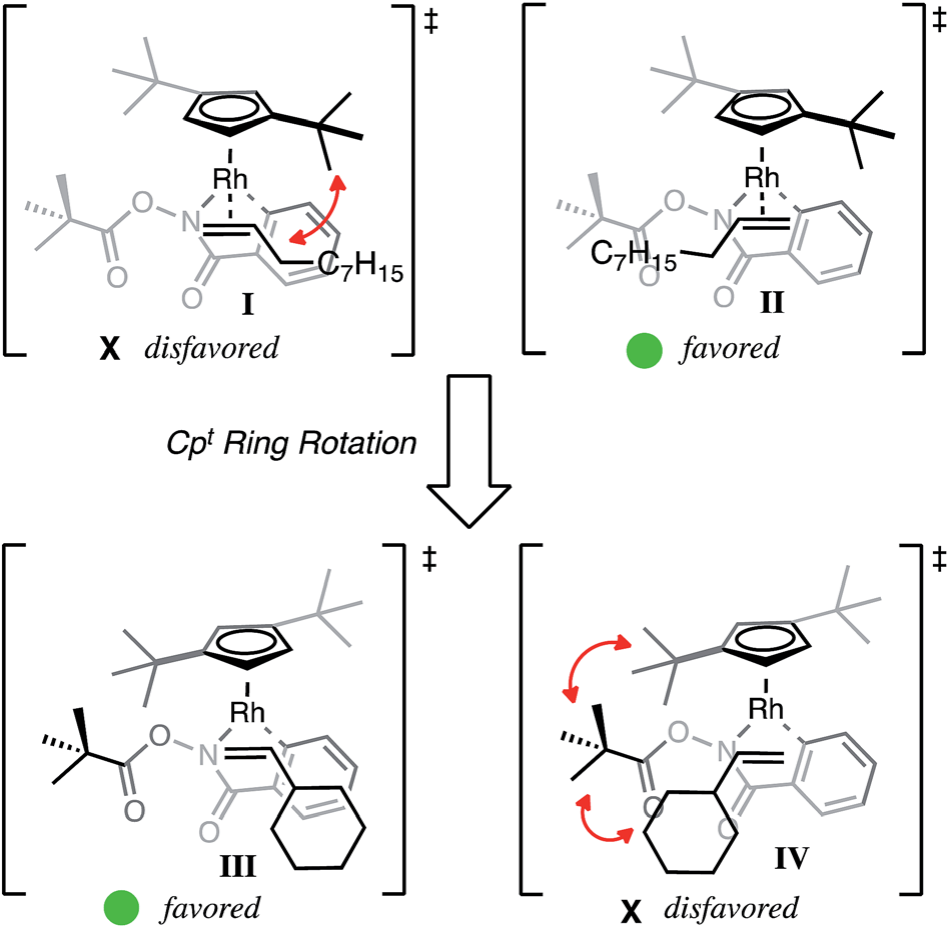
Rationale for selectivity.

However if the size of the alkene substituent is significantly increased, as in the case of vinyl cyclohexane, then Cp^*t*^ favors the opposite regioisomer. While certainly a puzzling result, we propose that the selectivity can be explained by Cp^*t*^ rotation such that the *t*-butyl groups both occupy the space above the metallacycle. Cp^*t*^ rotation gears the *O*-piv toward the alkene coordination site disfavoring alkene coordination to this side (**IV**) favoring the α-substituted product **3a**. At the same time, alkene coordination (**III**) with the cyclohexyl opposite the *O*-piv minimizes steric interactions enabling insertion of the large alkene and preferential formation of β-substituted product **3a′**. While not conclusive, the observation that cyclohexyl alkene reacts significantly slower than *n*-octyl alkene suggests that migratory insertion of the cyclohexyl alkene proceeds through a higher energy and potentially highly ordered transition state, such as Cp^*t*^ rotation.

## Conclusions

In conclusion, we found that sterically bulky di-*tert*-butyl cyclopentadienyl ligand (Cp^*t*^) is effective at increasing regioselectivity of alkene migratory insertion in the synthesis of dihydroisoquinolones by Rh(iii) C–H activation catalysis. In contrast to previous cases where Cp* delivers modest levels of regioselectivity of migratory insertion (with alkynes), Cp^*t*^ renders previously non-selective reactions (with alkenes) highly regioselective. Furthermore, ligand control (Cp* *vs.* Cp^*t*^) enables the highly selective synthesis of two regioisomeric products with vinylcyclohexanes. Finally, crystallographic evidence lends support for a possible explanation of the enhanced regioselectivity.
