# Correction: Ligand design for Rh(iii)-catalyzed C–H activation: an unsymmetrical cyclopentadienyl group enables a regioselective synthesis of dihydroisoquinolones

**DOI:** 10.1039/c6sc90080a

**Published:** 2016-12-13

**Authors:** Todd K. Hyster, Derek M. Dalton, Tomislav Rovis

**Affiliations:** a Department of Chemistry , Colorado State University , Fort Collins , Colorado 80523 , USA . Email: rovis@lamar.colostate.edu; b Department of Chemistry , Columbia University , 3000 Broadway , New York , NY 12007 , USA . Email: tr2504@columbia.edu

## Abstract

Correction for ‘Ligand design for Rh(iii)-catalyzed C–H activation: an unsymmetrical cyclopentadienyl group enables a regioselective synthesis of dihydroisoquinolones’ by Todd K. Hyster *et al.*, *Chem. Sci.*, 2015, **6**, 254–258.



## 

In the original article, Fig. 1 describes the coupling of *O*-pivaloyl benzhydroxamic acids with vinyl cyclohexane. Upon further analysis, the authors discovered that [RhCp*Cl_2_]_2_ provides lactam **4b** preferentially (11 : 1 rr and 90% yield) while [RhCp^*t*^Cl_2_]_2_ provides lactam **4a** (10 : 1 rr and 75% yield). The authors regret the error. A modified Fig. 1 is provided herein to clarify this error.

**Fig. 1:**
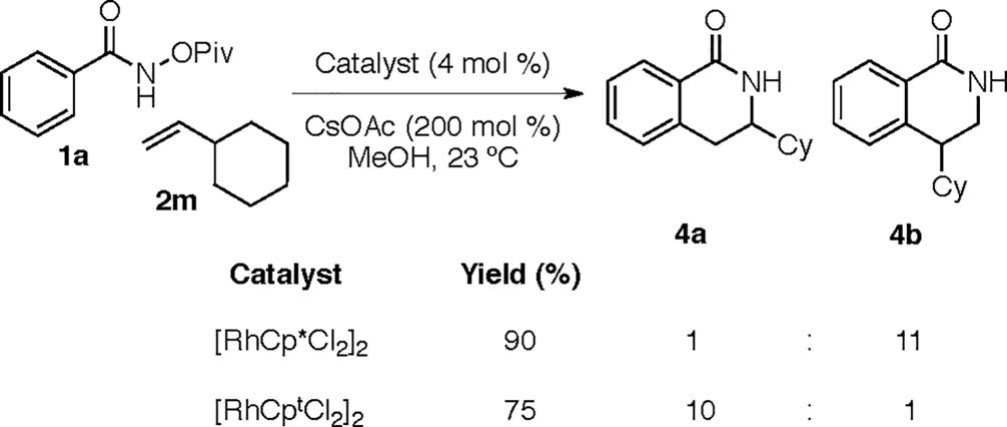
Impact of ligand on reaction of vinyl cyclohexane.

In addition, the authors wish to provide updated affiliation and contact details, and as such affiliation *b* has been added to the corresponding author of this article. Full details may be found herein.

The Royal Society of Chemistry apologises for these errors and any consequent inconvenience to authors and readers.

