# Correction: Ligand design for Rh(iii)-catalyzed C–H activation: an unsymmetrical cyclopentadienyl group enables a regioselective synthesis of dihydroisoquinolones

**DOI:** 10.1039/c8sc90195c

**Published:** 2018-10-15

**Authors:** Todd K. Hyster, Derek M. Dalton, Tomislav Rovis

**Affiliations:** a Department of Chemistry , Colorado State University , Fort Collins , Colorado 80523 , USA . Email: rovis@lamar.colostate.edu; b Department of Chemistry , Columbia University , 3000 Broadway , New York , NY 12007 , USA . Email: tr2504@columbia.edu

## Abstract

Correction for ‘Ligand design for Rh(iii)-catalyzed C–H activation: an unsymmetrical cyclopentadienyl group enables a regioselective synthesis of dihydroisoquinolones’ by Todd K. Hyster *et al.*, *Chem. Sci.*, 2015, **6**, 254–258.



## 

In the original manuscript, we reported that Cp^*t*^Rh(iii) complexes enable the highly regioselective coupling of simple alkenes with benzhydroxamate esters forming predominantly the products of 3-substituted dihydroisoquinolones. It has come to our attention that we misassigned the structures and the reaction provides 4-substituted dihydroisoquinolones (the product that we had initially assigned as the minor isomer has proven to be the major isomer). Thus, while the yields and regioselectivities as reported are unchanged, the assignment of the major isomer must be corrected.

Please see Table 1 below, which shows the correct isomer assignments. The information contained in Table 1 is a general example of the correct assignments that should be given, which covers all of the relevant experimental results and the tabulated data contained in the original article.

**Table 1:** General overview of the correct isomer assignments, which covers all of the relevant experimental results and the tabulated data in the original research article

We are grateful to Professor Dmitry Perekalin (Nesmeyanov Institute of Organoelement Compounds, Russian Academy of Sciences) for bringing this matter to our attention. The authors regret the error.

The original ESI was replaced by a correspondingly revised version on 9^th^ October 2018.

In addition, the authors wish to provide updated affiliation and contact details, and as such affiliation b has been added to the corresponding author of this article. Full details may be found herein.

The Royal Society of Chemistry apologises for these errors and any consequent inconvenience to authors and readers.

