# Supplementary material for: Ligand design for Rh(iii)-catalyzed C–H activation: an unsymmetrical cyclopentadienyl group enables a regioselective synthesis of dihydroisoquinolones
Source: Chem Sci. 2014 Oct 1;6(1):254–8. doi: 10.1039/c4sc02590c (PMC4256080; doi:10.1039/c4sc02590c)
Supplement: Supplementary file 1 [file SC-006-C4SC02590C-s001.pdf]

Electronic Supplementary Material (ESI) for Chemical Science.
This journal is © The Royal Society of Chemistry 2014

Supporting Information

Ligand Design for Rh(III)-Catalyzed C–H Activation: An Unsymmetrical Cyclopentadienyl Enables a Regioselective Synthesis of Dihydroisoquinolones

General Methods

All reactions were carried out in 1.5 dram sealed vials under an atmosphere of air without drying or degassing of the vial. HPLC grade MeOH was purchased from Fischer Scientific and used without further purification. All alkenes were purchased from commercial vendors and used without further purification. Column chromatography was performed on SiliCycle® Silica Flash® 40-63µm 60A. Thin Layer chromatography was performed on SiliCycle® 250 µm 60A plates. Visualization was accomplished with UV light (254 nm), $KMnO_4$, and CAM.

^{1}H NMR and ^{13}C NMR spectra were recorded on a Varian 300 and 400 MHz spectrometers at ambient temperature. ^{1}H NMR data are reported as the following: chemical shift in parts per million (δ, ppm) from chloroform ($CHCl_3$) taken as 7.26 ppm, integration, multiplicity (s=singlet, d=doublet, t=triplet, q=quartet, m=multiplet, dd=doublet of doublets) and coupling constant (Hz). ^{13}C NMR are reported as the following: chemical shifts are reported in ppm from $CDCl_3$ taken as 77.0 ppm. Mass spectra were obtained on a Fisons VG Autospec. Infrared spectra (IR) were obtained on Bruker Tensor 27 FT-IR spectrometer. The regioselectivities were determined by ^{1}H NMR and GC/MS. Absolute regiochemistry was determined by chemical shift in the ^{1}H NMR or by NOESY. **3m** was previous characterized by Fagnou.[5]

Catalyst Synthesis

$[RhCp^*Cl_2]_2$ and $[RhCp^‡Cl_2]_2$ were prepared from literature procedures.[1,2]

$[RhCp^tCl_2]_2$ was prepared from $RhCl_3•H_2O$ and Cp^tH, which can be accessed in one step from commercially available starting materials.[3] A procedure described by Kong and Jin was used to prepare the catalyst.[4] The catalyst is also now commercially available from Aldrich (RNI00147).

^{1}H NMR (400 MHz, $CDCl_3$) δ 5.64 (s, 2H), 5.35 (s, 1H), 1.33 (s, 18H); **^{13}C NMR** (100 MHz, $CDCl_3$) δ 108.67, 108.58, 82.43, 82.34, 81.24, 81.16, 31.1, 30.0.

Starting Amide Synthesis

All pivaloyl protected benzhydroxamic acids were prepared as described by Fagnou.[5] Protected hydroxamic acids were previously reported.[6,7,8,9]

---

[1] Fujita, K.; Takahashi, Y.; Owaki, M.; Yamamoto, K.; Yamaguchi, R. *Org. Lett.* **2004**, *6*, 2785.
[2] Gassman, P. G.; Mickelson, J. W.; Sowa, J. R., Jr *J. Am. Chem. Soc.* **1992**, *114*, 6942.
[3] Venier, C. G.; Casserly, E. W. *J. Am. Chem. Soc.* **1990**, *112*, 2808.
[4] Kong, Q.-A.; Jin, G.-X. *Chinese Journal of Applied Chemistry* **2013**, *18*, 322.
[5] Guimond, N.; Gorelsky, S. I.; Fagnou, K. *J. Am. Chem. Soc.* **2011**, *133*, 6449
[6] Wang, H.; Glorius, F. *Angew. Chem. Int. Ed.* **2012**, *51*, 7318
[7] Wang, H.; Grohmann, C.; Nimphius, C.; Glorius, F. *J. Am. Chem. Soc.* **2012**, *134,* 19592.
[8] Hyster, T. K.; Knörr, L.; Ward, T. R.; Rovis, T. *Science* **2012**, *338*, 500.
[9] Hyster, T. K.; Ruhl, K. E.; Rovis, T. *J. Am. Chem. Soc.* **2013**, *135*, 5364.

General Procedure

A 1.5 dram vial was charged with a stir bar, benzhydroxamic acid (0.2 mmol, 1 equiv), rhodium catalyst (0.002 mmol, 1 mol %), and CsOAc (0.4 mmol). After the solids were dissolved in MeOH (2 ml, 0.1 M), alkene (0.22 mmol, 1.1 equiv) was added and the vial was seal and allowed to stir for 16 hours. Upon completion, the crude mixture was transferred to a flask with excess DCM and condensed onto silica gel. The crude residue condensed on silica gel was purified via column chromatography. In the event that the purified compound was tainted with PivOH, the residues was dissolved in MeCN and extracted with hexanes (3x). The acetonitrile layer is concentrated *in vacuo* to yield the desired product.

Dihydroisoquinolone Synthesis Using Cp^t

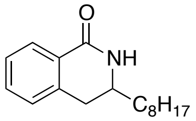

3-octyl-3,4-dihydroisoquinolin-1(2H)-one (3a): The crude reaction mixture was purified using a 2:1 Hexanes/EtOAc with 1% Et_3N. The product was isolated in 92% yield with 15:1 regioselectivity.

R_f (1:1 Hexanes/EtOAc) = 0.39.

^{1}H NMR (400 MHz, $CDCl_3$) δ 8.05-8.03 (m, 1H), 7.43 (td, J = 7.5, 0.5, 1H), 7.32 (t, J = 7.5, 1H), 7.18 (d, J = 7.5, 1H), 6.65 (s, br, 1H), 3.69 (dd, J = 12.4, 4.4, 1H), 3.38 (dd, J = 10.1, 6.2, 1H), 2.80 (t, J = 3.8, 1H), 1.29-1.18 (m, 13H), 0.8 (t, 2H, J = 6.4 Hz).

^{13}C NMR (100 MHz, $CDCl_3$) δ 166.2, 143.3, 132.0, 128.04, 128.00, 126.94, 126.92, 44.0, 37.9, 33.3, 31.8, 29.57, 29.45, 29.2, 27.3, 22.6, 14.1.

FTIR (cm^{-1}) 2923, 2853, 1670, 1475, 759.

HRMS (ESI) Calcd (M+1) 260.1936; found 260.2009.

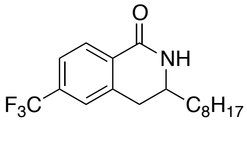

3-octyl-6-(trifluoromethyl)-3,4-dihydroisoquinolin-1(2*H*)-one (3b): The crude reaction mixture was purified using a 2:1 Hexanes/EtOAc with 1% Et_3N. The product was isolated in 50% yield with 19:1 regioselectivity.

R_f (1:1 Hexanes/EtOAc) = 0.45.

^{1}H NMR (400 MHz, $CDCl_3$) δ 8.15 (d, J = 8.1, 1H), 7.58 (dd, J = 8.1, 0.8, 1H), 7.44 (d, J = 0.5, 1H), 6.19 (d, J = 2.1, 1H), 3.71 (ddd, J = 12.5, 4.4, 1.3, 1H), 3.41 (ddd, J = 12.5, 4.5, 3.5, 1H), 2.88 (dt, J = 7.2, 3.6, 1H), 1.67 (ddd, J = 6.3, 5.7, 4.4, 2H), 1.30-1.23 (m, 13H), 0.85 (t, J = 6.9, 3H).

^{13}C NMR (100 MHz, $CDCl_3$) δ 164.6, 143.9, 128.8, 123.91, 123.87, 123.84, 43.7, 37.9, 33.1, 31.8, 29.45, 29.37, 29.2, 27.2, 22.6, 14.0.

FTIR (cm^{-1}) 2925, 2855, 1677, 1330, 1107, 1079.

HRMS (ESI) Calcd (M+1) 328.181; found 328.1889.

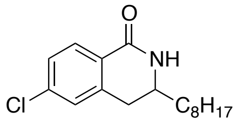

3-octyl-6-chloro-3,4-dihydroisoquinolin-1(2*H*)-one (3c): The crude reaction mixture was purified using a 2:1 Hexanes/EtOAc with 1% Et_3N. The product was isolated in 76% yield with 16:1 regioselectivity.

R_f (1:1 Hexanes/EtOAc) = 0.42.

^{1}H NMR (400 MHz, $CDCl_3$) δ 7.98 (d, J = 8.3, 1H), 7.30 (dd, J = 8.3, 2.0, 1H), 7.18 (d, J = 2.0, 1H), 6.42-6.41 (m, 1H), 3.67 (ddd, J = 12.5, 4.4, 1.3, 1H), 3.36 (dt, J = 12.4, 4.0, 1H), 2.78 (dd, J = 7.3, 3.6, 1H), 1.66 (dt, J = 12.0, 5.6, 2H), 1.29-1.24 (m, 13H), 0.86 (t, J = 6.8, 3H).

^{13}C NMR (100 MHz, $CDCl_3$) δ 165.3, 145.0, 138.1, 129.7, 127.3, 126.9, 126.5, 43.9, 37.8, 33.0, 31.8, 29.50, 29.39, 29.20, 27.2, 22.6, 14.0.

FTIR (cm^{-1}) 2923, 2853, 1678, 1596, 1467, 1090, 781.

HRMS (ESI) Calcd (M+1) 294.1546; found 294.1619.

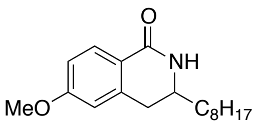

3-octyl-6-(methoxy)-3,4-dihydroisoquinolin-1(2*H*)-one (3d): The crude reaction mixture was purified using a 2:1 Hexanes/EtOAc with 1% Et_3N. The product was isolated in 70% yield with 14:1 regioselectivity.

$\mathbf{R_f}$ (1:1 Hexanes/EtOAc) = 0.27.

^{1}H NMR (400 MHz, $CDCl_3$) δ 7.99 (d, J = 8.6, 1H), 6.83 (dd, J = 8.6, 2.5, 1H), 6.67 (d, J = 2.5, 1H), 6.02 (d, J = 2.0, 1H), 3.83 (d, J = 4.2, 3H), 3.68-3.64 (m, 1H), 3.34 (dt, J = 8.3, 4.0, 1H), 2.75 (t, J = 3.4, 1H), 1.69-1.63 (m, 3H), 1.27-1.24 (m, 12H), 0.85 (t, J = 6.8, 3H).

^{13}C NMR (100 MHz, $CDCl_3$) δ 166.0, 162.5, 145.4, 130.2, 120.9, 112.3, 112.0, 55.3, 44.1, 38.3, 33.2, 31.8, 29.57, 29.44, 29.2, 27.4, 22.6, 14.0.

FTIR (cm^{-1}) 2923, 2852, 1678, 1605, 1474, 1265, 1248, 1156, 1031, 781.

HRMS (ESI) Calcd (M+1) 290.2044; found 290.2116.

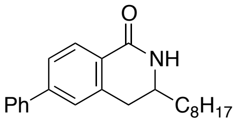

3-octyl-6-phenyl-3,4-dihydroisoquinolin-1(2*H*)-one (3e): The crude reaction mixture was purified using a 2:1 Hexanes/EtOAc with 1% Et_3N. The product was isolated in 75% yield with 14:1 regioselectivity.

$\mathbf{R_f}$ (1:1 Hexanes/EtOAc) = 0.39.

^{1}H NMR (400 MHz, $CDCl_3$) δ 8.11 (d, J = 8.0, 1H), 7.61-7.59 (m, 2H), 7.56 (dd, J = 8.0, 1.7, 1H), 7.47-7.43 (m, 2H), 7.40-7.37 (m, 2H), 6.23-6.22 (m, 1H), 3.76-3.72 (m, 1H), 3.44-3.40 (m, 1H), 2.88 (t, J = 3.6, 1H), 1.73-1.69 (m, 2H), 1.26 (d, J = 20.4, 12H), 0.85 (t, J = 8.2, 3H)

^{13}C NMR (100 MHz, $CDCl_3$) δ 165.9, 144.8, 143.7, 140.3, 128.84, 128.65, 128.0, 127.2, 126.8, 125.76, 125.63, 44.1, 38.2, 33.4, 31.8, 29.57, 29.45, 29.2, 27.4, 22.6, 14.0.

FTIR (cm^{-1}) 2923, 2852, 1679, 1609, 1473, 1332, 758, 732, 697.

HRMS (ESI) Calcd (M+1) 336.2249; found 336.2324.

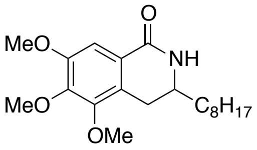

5,6,7-trimethoxy-3-octyl-3,4-dihydroisoquinolin-1(2*H*)-one (3f): The crude reaction mixture was purified using a 2:1 Hexanes/EtOAc with 1% Et_3N. The product was isolated in 95% yield with 15:1 regioselectivity.

R$_f$ (1:1 Hexanes/EtOAc) = 0.1.

^{1}H NMR (400 MHz, $CDCl_3$) δ 7.38 (s, 1H), 3.89 (s, 3H), 3.89 (d, J = 3.0, 4H), 3.87 (s, 3H), 3.58 (dd, J = 12.5, 4.2, 1H), 3.41 (dd, J = 5.3, 1.1, 1H), 3.01 (s, 1H), 1.42-1.39 (m, 2H), 1.23 (t, J = 7.0, 12H), 0.84 (t, J = 8.6 3H).

^{13}C NMR (100 MHz, $CDCl_3$) δ 165.8, 152.2, 149.5, 145.4, 130.5, 123.3, 106.4, 61.1, 60.7, 56.1, 43.1, 32.6, 31.81, 31.66, 29.47, 29.44, 29.2, 27.6, 22.6, 14.0.

FTIR (cm^{-1}) 2924, 2852, 1667, 1474, 1341, 1112, 1032.

HRMS (ESI) Calcd (M+1) 350.2253; found 350.2327.

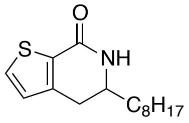

5-octyl-5,6-dihydrothieno[2,3-*c*]pyridin-7(4*H*)-one (3g): The crude reaction mixture was purified using a 2:1 Hexanes/EtOAc with 1% Et_3N. The product was isolated in 84% yield with 19:1 regioselectivity.

R$_f$ (1:1 Hexanes/EtOAc) = 0.47.

^{1}H NMR (400 MHz, $CDCl_3$) δ 7.30 (d, J = 1.9, 1H), 6.68 (d, J = 1.9, 1H), 6.29 (s, 1H), 3.64 (ddd, J = 12.3, 6.0, 2.6, 1H), 3.31 (ddd, J = 12.3, 7.3, 2.7, 1H), 3.00 (t, J = 6.8, 1H), 1.82-1.79 (m, 1H), 1.52 (dd, J = 14.3, 6.7, 1H), 1.37 (q, J = 7.4, 2H), 1.32-1.19 (m, 10H), 0.85 (t, J = 6.8, 3H)

^{13}C NMR (100 MHz, $CDCl_3$) δ 165.6, 163.0, 142.4, 114.3, 107.6, 45.7, 33.8, 31.8, 30.5, 29.47, 29.35, 29.17, 26.9, 22.6, 14.0.

FTIR (cm^{-1}) 2923, 2359, 1675, 1491, 1465, 1314, 1206, 1139, 732.

HRMS (ESI) Calcd (M+1) 266.15; found 266.1574.

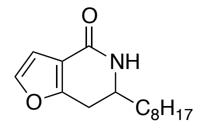

3-octyl-7-(trifluoromethyl)-3,4-dihydroisoquinolin-1(2*H*)-one (3h): The crude reaction mixture was purified using a 2:1 Hexanes/EtOAc with 1% Et_3N. The product was isolated in 88% yield with 19:1 regioselectivity.

R_f (1:1 Hexanes/EtOAc) = 0.27.

^{1}H NMR (400 MHz, $CDCl_3$) δ 7.47 (d, J = 5.0, 1H), 6.95 (d, J = 5.0, 1H), 6.26 (s, 1H), 3.68 (ddd, J = 12.3, 5.3, 2.3, 1H), 3.36 (ddd, J = 12.3, 6.3, 3.2, 1H), 2.93 (td, J = 5.9, 2.2, 1H), 1.73 (dd, J = 9.0, 4.5, 1H), 1.58 (dd, J = 9.0, 4.4, 1H), 1.26 (d, J = 14.0, 10H), 0.86 (t, J = 6.9, 3H).

^{13}C NMR (100 MHz, $CDCl_3$) δ 163.3, 149.4, 130.9, 126.2, 46.2, 35.3, 32.4, 31.8, 29.58, 29.42, 29.2, 27.1, 22.6, 14.0.

FTIR (cm^{-1}) 2922, 2852, 1676, 1475, 1432, 1333, 1147, 773, 714.

HRMS (ESI) Calcd (M+1) 250.1729; found 250.1810.

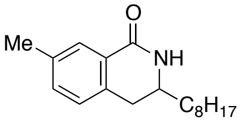

3-octyl-7-methyl-3,4-dihydroisoquinolin-1(2*H*)-one (3i): The crude reaction mixture was purified using a 2:1 Hexanes/EtOAc with 1% Et_3N. The product was isolated in 67% yield with 15:1 regioselectivity.

R_f (1:1 Hexanes/EtOAc) = 0.39.

^{1}H NMR (400 MHz, $CDCl_3$) δ 7.86 (s, 1H), 7.26-7.21 (m, 2H), 7.08 (d, J = 7.7, 1H), 6.34 (s, 1H), 3.66 (ddd, J = 12.3, 4.4, 1.2, 1H), 3.36-3.33 (m, 1H), 2.77 (d, J = 3.6, 1H), 2.34 (d, J = 12.2, 4H), 1.62 (t, J = 6.9, 2H), 1.23 (s, 12H), 0.85 (t, J = 6.8, 3H).

^{13}C NMR (100 MHz, $CDCl_3$) δ 166.3, 140.3, 136.6, 132.8, 128.4, 127.8, 126.9, 44.2, 37.5, 33.3, 31.8, 29.58, 29.45, 29.2, 27.3, 22.6, 21.0, 14.0.

FTIR (cm^{-1}) 2922, 2852, 1680, 1613, 1479, 1456, 1326, 821, 760.

HRMS (ESI) Calcd (M+1) 274.2093; found 274.2174.

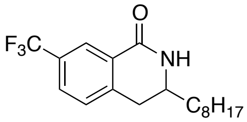

3-octyl-7-(trifluoromethyl)-3,4-dihydroisoquinolin-1(2*H*)-one (3j): The crude reaction mixture was purified using a 2:1 Hexanes/EtOAc with 1% Et_3N. The product was isolated in 80% yield with 19:1 regioselectivity.

R_f (1:1 Hexanes/EtOAc) = 0.27.

^{1}H NMR (400 MHz, $CDCl_3$) δ 8.34 (d, J = 0.4, 1H), 7.70-7.68 (m, 1H), 7.32 (d, J = 8.0, 1H), 5.97-5.96 (m, 1H), 3.74-3.70 (m, 1H), 3.41 (dt, J = 12.5, 3.9, 1H), 2.91-2.88 (m, 1H), 1.72-1.64 (m, 2H), 1.27-1.16 (m, 13H), 0.87-0.80 (m, 3H).

^{13}C NMR (100 MHz, $CDCl_3$) δ 164.4, 146.8, 129.5, 128.67, 128.50, 128.47, 128.43, 127.7, 125.46, 125.42, 125.38, 98.5, 93.3, 43.8, 37.9, 33.2, 31.8, 29.67, 29.48, 29.39, 29.2, 27.3, 22.6, 14.0.

FTIR (cm^{-1}) 2924, 2854, 1678, 1621, 1483, 1318, 1160, 1100, 838.

HRMS (ESI) Calcd (M+1) 328.181; found 328.1890.

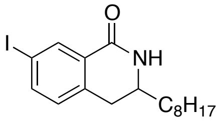

3-octyl-7-iodo-3,4-dihydroisoquinolin-1(2*H*)-one (3k): The crude reaction mixture was purified using a 2:1 Hexanes/EtOAc with 1% Et_3N. The product was isolated in 85% yield with 16:1 regioselectivity. The regioselectivity for the C–H activation event is 1:1 and are inseperable.

R_f (1:1 Hexanes/EtOAc) = 0.20.

¹H NMR (400 MHz, $CDCl_3$) δ See spectra for 1:1 ratio of C–H activation regioisomers.

¹³C NMR (100 MHz, $CDCl_3$) δ 165.2, 164.6, 146.2, 143.1, 142.6, 140.7, 136.9, 129.8, 129.5, 128.9, 128.53, 128.40, 98.7, 91.8, 43.9, 42.3, 41.5, 37.5, 33.1, 31.82, 31.77, 30.7, 29.51, 29.44, 29.41, 29.37, 29.21, 29.20, 27.6, 27.2, 22.61, 22.59, 14.06, 14.04.

FTIR (cm^{-1}) 2921, 2851, 1678, 1587, 1478, 1323, 754.

HRMS (ESI) Calcd (M+1) 386.0903; found 386.0981.

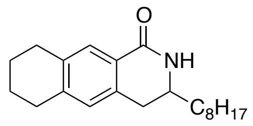

3-octyl-3,4,6,7,8,9-hexahydrobenzo[*g*]isoquinolin-1(2*H*)-one (3l): The crude reaction mixture was purified using a 2:1 Hexanes/EtOAc with 1% Et_3N. The product was isolated in 79% yield with 18:1 regioselectivity.

$\mathbf{R_f}$ (1:1 Hexanes/EtOAc) = 0.20.

¹H NMR (400 MHz, $CDCl_3$) δ 7.74 (s, 1H), 6.87 (s, 1H), 6.37 (d, J = 0.7, 1H), 3.66-3.61 (m, 1H), 3.34-3.31 (m, 1H), 2.77 (s, 4H), 1.78 (t, J = 2.9, 4H), 1.62 (t, J = 6.9, 2H), 1.24 (d, J = 7.1, 12H), 0.85 (t, J = 6.9, 3H).

¹³C NMR (100 MHz, $CDCl_3$) δ 166.5, 141.8, 140.2, 136.0, 128.6, 127.3, 125.3, 44.2, 37.5, 33.3, 31.8, 29.64, 29.58, 29.50, 29.45, 29.2, 28.9, 27.4, 23.00, 22.91, 22.6, 14.1.

FTIR (cm^{-1}) 2922, 2853, 1677, 1614, 1476, 1328, 729

HRMS (ESI) Calcd (M+1) 314.2406; found 314.2483.

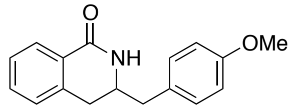

3-(4-methoxybenzyl)-3,4-dihydroisoquinolin-1(2H)-one (3n): The crude reaction mixture was purified using a 2:1 Hexanes/EtOAc with 1% Et_3N. The product was isolated in 68% yield with 9.4:1 regioselectivity.

R_f (1:1 Hexanes/EtOAc) = 0.23.

^{1}H NMR (400 MHz, $CDCl_3$) δ 8.09 (d, J = 7.5, 1H), 7.41 (td, J = 7.4, 1.3, 1H), 7.37-7.33 (m, 1H), 7.07 (t, J = 5.5, 1H), 7.03 (d, J = 8.5, 2H), 6.85-6.80 (m, 2H), 6.13-6.13 (m, 1H), 3.78 (s, 3H), 3.57 (dd, J = 12.4, 4.1, 1H), 3.27 (ddd, J = 12.4, 4.4, 2.9, 1H), 3.03-3.01 (m, 1H), 2.93-2.83 (m, 2H).

^{13}C NMR (100 MHz, $CDCl_3$) δ 166.0, 158.2, 142.4, 132.2, 131.0, 130.13, 130.02, 128.2, 127.9, 127.22, 127.09, 114.3, 113.9, 98.7, 55.2, 42.9, 40.1, 38.9.

FTIR (cm^{-1}) 2923, 1677, 1511, 1475, 1245, 1177, 1033, 819.

HRMS (ESI) Calcd (M+1) 268.1259; found 268.1333.

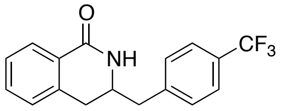

3-(4-(trifluoromethyl)benzyl)-3,4-dihydroisoquinolin-1(2H)-one (3o): The crude reaction mixture was purified using a 2:1 Hexanes/EtOAc with 1% Et_3N. The product was isolated in 70% yield with 5.5:1 regioselectivity.

R_f (1:1 Hexanes/EtOAc) = 0.35.

^{1}H NMR (400 MHz, $CDCl_3$) δ 8.09 (dd, J = 7.3, 1.8, 1H), 7.52 (t, J = 6.0, 2H), 7.43-7.34 (m, 2H), 7.21 (d, J = 8.0, 2H), 7.00-6.98 (m, 1H), 6.29-6.28 (m, 1H), 3.63 (ddd, J = 12.5, 4.1, 0.9, 1H), 3.26 (ddd, J = 12.6, 4.8, 2.7, 1H), 3.12-3.05 (m, 1H), 3.00 (t, J = 8.0, 2H).

^{13}C NMR (100 MHz, $CDCl_3$) δ 165.9, 143.1, 141.6, 132.2, 129.4, 128.3, 128.0, 127.5, 127.1, 125.48, 125.44, 125.41, 43.1, 39.75, 39.61.

FTIR (cm^{-1}) 1667, 1389, 1107, 1066, 752, 729.

HRMS (ESI) Calcd (M+1) 306.1027; found 306.1088.

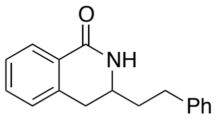

3-phenethyl-3,4-dihydroisoquinolin-1(2H)-one (3p): The crude reaction mixture was purified using a 2:1 Hexanes/EtOAc with 1% Et_3N. The product was isolated in 95% yield with 14:1 regioselectivity.

R_f (1:1 Hexanes/EtOAc) = 0.23.

^{1}H NMR (400 MHz, $CDCl_3$) δ 8.07 (dd, J = 7.7, 1.4, 1H), 7.47-7.43 (m, 1H), 7.35 (td, J = 7.6, 1.2, 1H), 7.29-7.25 (m, 2H), 7.19-7.14 (m, 4H), 6.35-6.34 (m, 1H), 3.73 (ddd, J = 12.5, 4.3, 0.7, 1H), 3.43 (ddd, J = 12.5, 4.6, 3.2, 1H), 2.87 (dt, J = 7.3, 3.7, 1H), 2.75-2.68 (m, 1H), 2.61 (ddd, J = 14.0, 9.0, 6.9, 1H), 2.06-1.99 (m, 2H).

^{13}C NMR (100 MHz, $CDCl_3$) δ 166.0, 142.7, 141.3, 132.1, 128.59, 128.47, 128.25, 128.20, 128.14, 128.11, 128.08, 127.17, 127.04, 126.6, 126.0, 125.6, 44.0, 37.4, 34.8, 33.4, 31.4.

FTIR (cm^{-1}) 1677, 1475, 1334, 1157, 751, 699.

HRMS (ESI) Calcd (M+1) 252.131; found 252.1375.

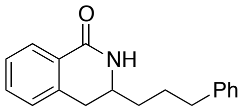

3-(3-phenylpropyl)-3,4-dihydroisoquinolin-1(2*H*)-one (3q): The crude reaction mixture was purified using a 2:1 Hexanes/EtOAc with 1% Et_3N. The product was isolated in 85% yield with 8:1 regioselectivity.

R_f (1:1 Hexanes/EtOAc) = 0.29.

^{1}H NMR (400 MHz, $CDCl_3$) δ 8.05-8.03 (m, 1H), 7.42 (td, J = 7.5, 1.3, 1H), 7.33 (t, J = 7.2, 1H), 7.27-7.23 (m, 1H), 7.18-7.12 (m, 4H), 6.51 (d, J = 1.7, 1H), 3.68 (dd, J = 12.4, 4.3, 1H), 3.35 (dt, J = 12.2, 3.9, 1H), 2.82 (t, J = 3.3, 1H), 2.59 (t, J = 6.9, 2H), 1.75-1.68 (m, 3H), 1.61 (dtd, J = 12.5, 6.7, 3.2, 1H).

^{13}C NMR (100 MHz, $CDCl_3$) δ 166.2, 142.9, 141.9, 132.1, 128.33, 128.31, 128.27, 128.11, 127.94, 127.07, 127.01, 125.8, 44.1, 37.8, 35.8, 32.9, 29.2.

FTIR (cm^{-1}) 2923, 2854, 1677, 1603, 1475, 1156, 730, 699.

HRMS (ESI) Calcd (M+1) 265.1467; found 266.1532.

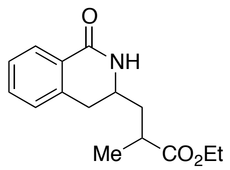

ethyl 2-methyl-3-(1-oxo-1,2,3,4-tetrahydroisoquinolin-3-yl)propanoate (3r): The crude reaction mixture was purified using a 2:1 Hexanes/EtOAc with 1% Et_3N. The product was isolated in 92% yield with 7.2:1 regioselectivity.

$\mathbf{R_f}$ (1:1 Hexanes/EtOAc) = 0.29.

^{1}H NMR is a mixture of diastereomers. See Spectra.

^{13}C NMR (100 MHz, $CDCl_3$) δ 176.3, 175.9, 165.92, 165.78, 142.6, 141.9, 132.4, 132.0, 128.34, 128.16, 128.06, 127.92, 127.46, 127.37, 127.27, 126.8, 60.50, 60.47, 45.1, 43.3, 37.6, 37.4, 37.10, 37.07, 35.8, 18.01, 17.95, 14.30, 14.24.

FTIR (cm^{-1}) 2924, 1727, 1677, 1476, 1162, 760

HRMS (ESI) Calcd (M+1) 262.1365; found 262.1430.

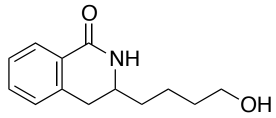

3-(4-hydroxybutyl)-3,4-dihydroisoquinolin-1(2*H*)-one (3s): The crude reaction mixture was purified using a 19:1 DCM/MeOH. The product was isolated in 80% yield with 12:1 regioselectivity.

$\mathbf{R_f}$ (19:1 DCM/MeOH) = 0.1.

^{1}H NMR (400 MHz, $CDCl_3$) δ 8.05-8.03 (m, 1H), 7.44 (td, J = 7.5, 1.3, 1H), 7.33 (td, J = 7.5, 0.9, 1H), 7.18 (d, J = 7.6, 1H), 6.39 (t, J = 0.6, 1H), 3.72-3.68 (m, 1H), 3.61 (t, J = 6.3, 2H), 3.38 (ddd, J = 12.4, 4.4, 3.3, 1H), 2.82 (dd, J = 7.1, 3.7, 1H), 1.73-1.66 (m, 2H), 1.59-1.51 (m, 3H), 1.50-1.34 (m, 2H).

^{13}C NMR (100 MHz, $CDCl_3$) δ 166.1, 143.0, 132.1, 128.11, 127.94, 127.07, 127.00, 62.5, 44.0, 37.9, 33.2, 32.6, 23.6.

FTIR (cm^{-1}) 3300, 2927, 2856, 1678, 1476, 1335, 762.

HRMS (ESI) Calcd (M+1) 220.1259; found 220.1324.

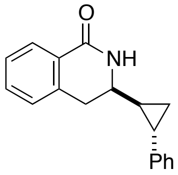

(*R*)-3-((1*S*,2*S*)-2-phenylcyclopropyl)-3,4-dihydroisoquinolin-1(2*H*)-one (3t):. The crude reaction mixture was purified using a 40:1 Toluene/iPrOH. The product was isolated in 93% yield with 11:1 regioselectivity.

$\mathbf{R_f}$ (1:1 Hexanes/EtOAc) = 0.27.

^{1}H NMR (400 MHz, $CDCl_3$) is a mixture of diastereomers. See Spectra.

^{13}C NMR (100 MHz, $CDCl_3$) δ 166.2, 166.0, 142.18, 142.02, 141.45, 141.44, 137.8, 132.41, 132.31, 129.0, 128.40, 128.29, 128.22, 128.19, 128.15, 128.06, 127.37, 127.31, 126.3, 126.04, 125.86, 125.78, 125.3, 45.5, 45.2, 42.74, 42.67, 25.67, 25.47, 23.2, 22.1, 21.4, 15.9, 13.8.

FTIR (cm^{-1}) 1664, 1603, 1496, 1336, 111, 751, 697.

HRMS (ESI) Calcd (M+1) 264.131; found 264.1372.

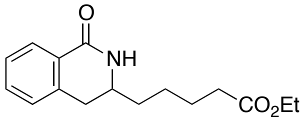

ethyl 5-(1-oxo-1,2,3,4-tetrahydroisoquinolin-3-yl)pentanoate (3u): The crude reaction mixture was purified using a 40:1 Toluene/iPrOH. The product was isolated in 89% yield with 14:1 regioselectivity.

$\mathbf{R_f}$ (1:1 Hexanes/EtOAc) = 0.16.

^{1}H NMR (400 MHz, $CDCl_3$) δ 8.04 (dd, J = 7.7, 0.9, 1H), 7.43 (td, J = 7.5, 1.3, 1H), 7.34-7.31 (m, 1H), 7.17 (d, J = 7.5, 1H), 6.56 (dd, J = 1.3, 0.4, 1H), 4.09 (q, J = 7.1, 2H), 3.69 (ddd, J =

12.4, 4.3, 0.8, 1H), 3.36 (ddd, J = 12.4, 4.4, 3.5, 1H), 2.81 (dd, J = 7.2, 3.7, 1H), 2.26 (t, J = 7.4, 2H), 1.71-1.58 (m, 2H), 1.45-1.29 (m, 2H), 1.22 (dd, J = 4.8, 3H)

^{13}C NMR (100 MHz, $CDCl_3$) δ 173.5, 166.1, 142.9, 132.1, 128.10, 127.98, 127.05, 126.95, 60.2, 44.0, 37.6, 34.1, 33.0, 26.7, 24.8, 14.2.

FTIR (cm^{-1}) 2931, 1728, 1678, 1476, 1178, 1031, 761.

HRMS (ESI) Calcd (M+1) 276.1516; found 276.1588.

HRMS (ESI) Calcd (M+1) 206.1103; found 206.1167.

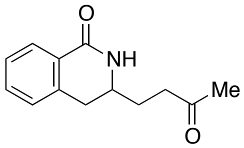

3-(3-oxobutyl)-3,4-dihydroisoquinolin-1(2*H*)-one (3v): The product was isolated in 84% yield. The crude reaction mixture was purified using a 40:1 Toluene/iPrOH. The product was isolated with 14:1 regioselectivity.

R_f (1:1 Hexanes/EtOAc) = 0.10.

^{1}H NMR (400 MHz, $CDCl_3$) δ 8.04 (d, J = 7.7, 1H), 7.44 (td, J = 7.5, 1.0, 1H), 7.34 (t, J = 7.5, 1H), 7.16-7.14 (m, 1H), 6.63 (s, 1H), 3.71 (dd, J = 12.5, 4.4, 1H), 3.35 (ddd, J = 12.5, 4.6, 2.9, 1H), 2.91-2.86 (m, 1H), 2.42 (t, J = 7.2, 2H), 1.95 (qd, J = 7.3, 3.5, 2H).

^{13}C NMR (100 MHz, $CDCl_3$) δ 207.9, 165.9, 141.9, 132.1, 128.22, 128.15, 127.34, 127.17, 44.4, 40.6, 36.6, 30.0, 27.0.

FTIR (cm^{-1}) 1709, 1678, 1603, 1475, 1334, 1160, 763.

HRMS (ESI) Calcd (M+1) 218.1103; found 218.1172.

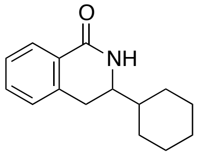

3-cyclohexyl-3,4-dihydroisoquinolin-1(2*H*)-one (4a): The crude reaction mixture was purified using a 40:1 Toluene/iPrOH. The product was isolated in

90% yield with 11:1 regioselectivity.

R$_f$ (1:1 Hexanes/EtOAc) = 0.43.

^{1}H NMR (400 MHz, $CDCl_3$) δ 8.03 (d, J = 7.7, 1H), 7.41 (t, J = 7.4, 1H), 7.31 (t, J = 7.5, 1H), 7.18 (d, J = 7.5, 1H), 6.06 (s, 1H), 3.45 (d, J = 6.9, 1H), 2.90 (d, J = 8.1, 2H), 1.77 (d, J = 9.7, 4H), 1.69-1.46 (m, 3H), 1.24-1.03 (m, 5H).

^{13}C NMR (100 MHz, $CDCl_3$) δ

FTIR (cm^{-1}) 2918, 2850, 1667, 1462, 756, 736.

HRMS (ESI) Calcd (M+1) 230.1467; found 230.1541.

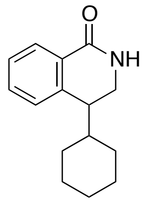

4-cyclohexyl-3,4-dihydroisoquinolin-1(2*H*)-one (4b): The crude reaction mixture was purified using a 40:1 Toluene/iPrOH. The product was isolated in 75% yield with 10:1 regioselectivity.

R$_f$ (1:1 Hexanes/EtOAc) = 0.26.

^{1}H NMR (400 MHz, $CDCl_3$) δ 8.03 (dd, J = 7.7, 1.1, 1H), 7.42 (td, J = 7.5, 1.4, 1H), 7.33 (td, J = 7.5, 1.1, 1H), 7.14 (d, J = 7.6, 1H), 6.04 (td, J = 1.3, 0.7, 1H), 3.65-3.54 (m, 2H), 2.49-2.47 (m, 1H), 1.87-1.44 (m, 8H), 1.24-0.94 (m, 5H).

^{13}C NMR (100 MHz, $CDCl_3$) δ 166.1, 141.7, 131.4, 128.49, 128.38, 127.9, 127.0, 43.9, 41.9, 39.4, 31.3, 31.0, 26.35, 26.30, 26.21.

FTIR (cm^{-1}) 2921, 2850, 1667, 1475, 1334, 761.

HRMS (ESI) Calcd (M+1) 230.1468; found 230.1540.

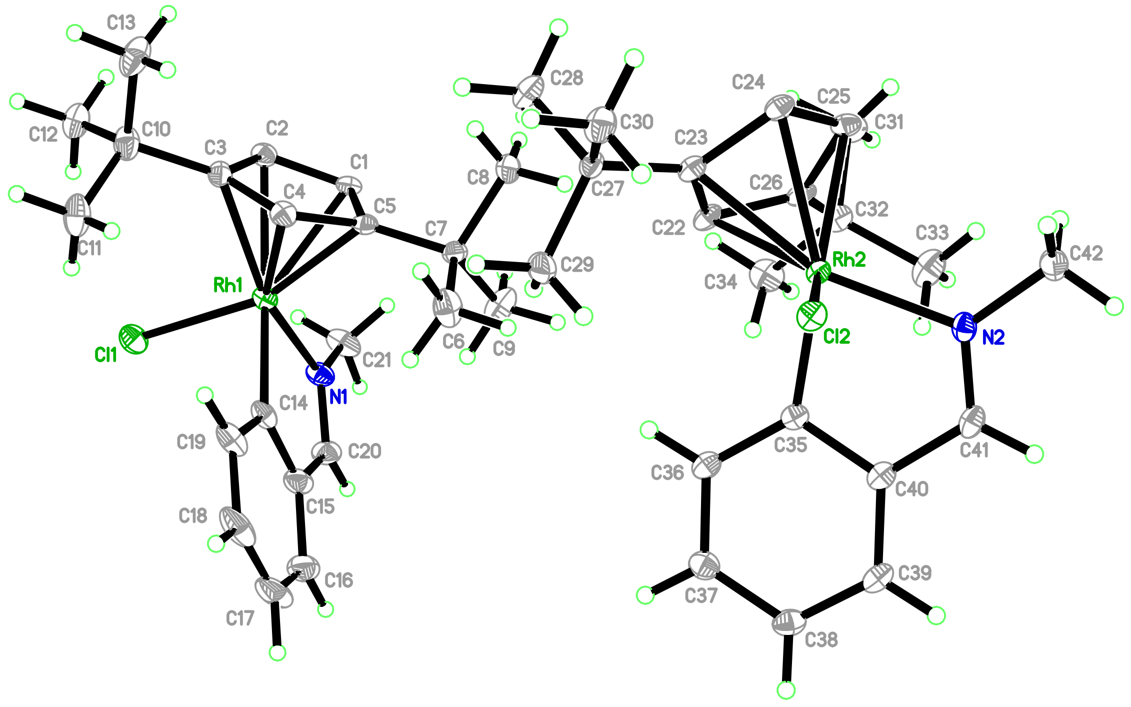

Table 1. Crystal data and structure refinement for Rovis167_0m.

Identification code	rovis167_0m	
Empirical formula	$C_{21} H_{29} Cl N Rh$	
Formula weight	433.81	
Temperature	120(2) K	
Wavelength	0.71073 Å	
Crystal system	Triclinic	
Space group	*P*-1	
Unit cell dimensions	*a* = 11.7956(12) Å	α = 85.713(5)°.
	b = 12.3940(12) Å	β = 81.671(6)°.
	c = 13.9527(14) Å	γ = 80.615(5)°.
Volume	1988.5(3) Å^3	

Z	4
Density (calculated)	1.449 Mg/m^3
Absorption coefficient	0.995 mm^{-1}
F_{000}	896
Crystal size	0.07 x 0.06 x 0.05 mm^3
Theta range for data collection	1.67 to 30.58°.
Index ranges	$-16 \le h \le 16$, $-17 \le k \le 17$, $-19 \le l \le 19$
Reflections collected	44475
Independent reflections	12069 [R_{int} = 0.0578]
Completeness to theta = 30.58°	98.8 %
Absorption correction	Semi-empirical from equivalents
Max. and min. transmission	0.9519 and 0.9318
Refinement method	Full-matrix least-squares on F^2
Data / restraints / parameters	12069 / 0 / 447
Goodness-of-fit on F^2	1.042
Final R indices [I>2sigma(I)]	R1 = 0.0519, wR2 = 0.1073
R indices (all data)	R1 = 0.1003, wR2 = 0.1324
Largest diff. peak and hole	2.566 and -1.227 e.$Å^{-3}$

Table 2. Atomic coordinates (x 10^4) and equivalent isotropic displacement parameters ($Å^2$x 10^3) for Rovis167_0m. U(eq) is defined as one third of the trace of the orthogonalized U^{ij} tensor.

	x	y	z	U(eq)
C(1)	8631(3)	8306(3)	2697(3)	22(1)
C(2)	9356(4)	8521(3)	3360(3)	26(1)
C(3)	9523(4)	7552(3)	4014(3)	26(1)
C(4)	8943(4)	6742(3)	3702(3)	25(1)
C(5)	8347(3)	7202(3)	2904(3)	23(1)
C(6)	7414(5)	5552(3)	2770(3)	41(1)
C(7)	7752(3)	6601(3)	2257(3)	24(1)
C(8)	8616(4)	6302(5)	1357(3)	48(1)
C(9)	6675(4)	7324(4)	1961(4)	45(1)
C(10)	10347(4)	7365(4)	4776(3)	35(1)
C(11)	9819(5)	6729(4)	5669(3)	45(1)
C(12)	10627(4)	8448(4)	5056(4)	40(1)
C(13)	11473(4)	6675(4)	4324(4)	49(1)
C(14)	6434(4)	7246(3)	4877(3)	28(1)
C(15)	5277(4)	7759(4)	4847(3)	33(1)
C(16)	4335(5)	7261(4)	5301(4)	44(1)
C(17)	4539(6)	6262(5)	5782(4)	57(2)
C(18)	5673(6)	5745(4)	5825(3)	51(2)

C(19)	6592(5)	6241(3)	5395(3)	36(1)
C(20)	5169(4)	8795(4)	4311(3)	33(1)
C(21)	6109(4)	10168(4)	3374(3)	40(1)
C(22)	8015(3)	3268(3)	350(3)	23(1)
C(23)	8880(3)	2355(3)	491(3)	22(1)
C(24)	9316(3)	1912(3)	-452(3)	23(1)
C(25)	8691(3)	2523(3)	-1146(3)	23(1)
C(26)	7848(3)	3380(3)	-655(3)	23(1)
C(27)	9402(3)	2053(3)	1426(3)	22(1)
C(28)	10241(4)	2879(4)	1483(3)	35(1)
C(29)	8467(4)	2133(4)	2308(3)	34(1)
C(30)	10081(4)	898(3)	1432(3)	32(1)
C(31)	7982(4)	4930(3)	-1867(3)	37(1)
C(32)	7125(4)	4345(3)	-1147(3)	26(1)
C(33)	6249(4)	3964(4)	-1685(3)	39(1)
C(34)	6503(4)	5143(3)	-390(3)	36(1)
C(35)	5812(3)	2068(3)	765(3)	20(1)
C(36)	5501(3)	2465(3)	1691(3)	24(1)
C(37)	4393(4)	2457(3)	2175(3)	27(1)
C(38)	3549(4)	2056(3)	1757(3)	28(1)
C(39)	3820(4)	1682(3)	844(3)	26(1)
C(40)	4941(3)	1677(3)	350(3)	22(1)
C(41)	5326(4)	1240(3)	-584(3)	23(1)

C(42)	6857(4)	814(3)	-1879(3)	29(1)
Cl(2)	7575(1)	-14(1)	680(1)	23(1)
Cl(1)	7467(1)	9110(1)	5614(1)	35(1)
N(1)	6124(3)	9110(3)	3912(2)	28(1)
N(2)	6383(3)	1264(2)	-942(2)	22(1)
Rh(2)	7401(1)	1815(1)	-40(1)	17(1)
Rh(1)	7634(1)	8124(1)	4168(1)	23(1)

Table 3. Bond lengths [Å] and angles [°] for Rovis167_0m.

C(1)-C(2)	1.413(6)
C(1)-C(5)	1.460(5)
C(1)-Rh(1)	2.228(4)
C(2)-C(3)	1.458(5)
C(2)-Rh(1)	2.284(4)
C(3)-C(4)	1.429(6)
C(3)-C(10)	1.523(6)
C(3)-Rh(1)	2.210(4)
C(4)-C(5)	1.435(5)
C(4)-Rh(1)	2.181(4)
C(5)-C(7)	1.522(6)
C(5)-Rh(1)	2.164(4)
C(6)-C(7)	1.519(5)
C(7)-C(9)	1.521(6)
C(7)-C(8)	1.526(6)
C(10)-C(12)	1.524(6)
C(10)-C(11)	1.534(6)
C(10)-C(13)	1.536(7)
C(14)-C(19)	1.392(5)
C(14)-C(15)	1.414(6)
C(14)-Rh(1)	2.025(4)
C(15)-C(16)	1.406(6)
C(15)-C(20)	1.434(6)
C(16)-C(17)	1.367(8)
C(17)-C(18)	1.394(8)
C(18)-C(19)	1.374(7)
C(20)-N(1)	1.289(5)
C(21)-N(1)	1.460(5)
C(22)-C(23)	1.418(5)
C(22)-C(26)	1.436(6)
C(22)-Rh(2)	2.180(4)
C(23)-C(24)	1.454(6)
C(23)-C(27)	1.519(5)
C(23)-Rh(2)	2.206(4)
C(24)-C(25)	1.404(5)
C(24)-Rh(2)	2.270(4)
C(25)-C(26)	1.469(6)
C(25)-Rh(2)	2.241(4)
C(26)-C(32)	1.530(5)
C(26)-Rh(2)	2.175(3)
C(27)-C(30)	1.522(5)

C(27)-C(29)	1.527(6)	C(1)-C(2)-C(3)	107.2(3)
C(27)-C(28)	1.548(5)	C(1)-C(2)-Rh(1)	69.6(2)
C(31)-C(32)	1.546(5)	C(3)-C(2)-Rh(1)	68.3(2)
C(32)-C(33)	1.513(6)	C(4)-C(3)-C(2)	108.0(3)
C(32)-C(34)	1.525(6)	C(4)-C(3)-C(10)	125.7(4)
C(35)-C(36)	1.395(5)	C(2)-C(3)-C(10)	125.5(4)
C(35)-C(40)	1.414(5)	C(4)-C(3)-Rh(1)	69.9(2)
C(35)-Rh(2)	2.031(4)	C(2)-C(3)-Rh(1)	73.9(2)
C(36)-C(37)	1.382(6)	C(10)-C(3)-Rh(1)	130.1(3)
C(37)-C(38)	1.397(6)	C(3)-C(4)-C(5)	108.9(3)
C(38)-C(39)	1.365(6)	C(3)-C(4)-Rh(1)	72.1(2)
C(39)-C(40)	1.398(6)	C(5)-C(4)-Rh(1)	70.1(2)
C(40)-C(41)	1.432(6)	C(4)-C(5)-C(1)	106.4(3)
C(41)-N(2)	1.279(5)	C(4)-C(5)-C(7)	127.2(3)
C(42)-N(2)	1.459(5)	C(1)-C(5)-C(7)	125.3(3)
Cl(2)-Rh(2)	2.3995(9)	C(4)-C(5)-Rh(1)	71.4(2)
Cl(1)-Rh(1)	2.4045(12)	C(1)-C(5)-Rh(1)	73.0(2)
N(1)-Rh(1)	2.050(3)	C(7)-C(5)-Rh(1)	130.2(3)
N(2)-Rh(2)	2.079(3)	C(6)-C(7)-C(9)	109.3(4)
		C(6)-C(7)-C(5)	111.0(3)
C(2)-C(1)-C(5)	109.5(3)	C(9)-C(7)-C(5)	110.7(3)
C(2)-C(1)-Rh(1)	74.0(2)	C(6)-C(7)-C(8)	108.5(4)
C(5)-C(1)-Rh(1)	68.2(2)	C(9)-C(7)-C(8)	109.7(4)

C(5)-C(7)-C(8)	107.6(3)	C(24)-C(23)-C(27)	126.6(3)
C(3)-C(10)-C(12)	111.0(4)	C(22)-C(23)-Rh(2)	70.1(2)
C(3)-C(10)-C(11)	109.9(4)	C(24)-C(23)-Rh(2)	73.4(2)
C(12)-C(10)-C(11)	110.8(4)	C(27)-C(23)-Rh(2)	130.9(3)
C(3)-C(10)-C(13)	107.2(4)	C(25)-C(24)-C(23)	108.3(3)
C(12)-C(10)-C(13)	108.9(4)	C(25)-C(24)-Rh(2)	70.8(2)
C(11)-C(10)-C(13)	109.0(4)	C(23)-C(24)-Rh(2)	68.7(2)
C(19)-C(14)-C(15)	116.7(4)	C(24)-C(25)-C(26)	108.4(3)
C(19)-C(14)-Rh(1)	129.4(4)	C(24)-C(25)-Rh(2)	73.0(2)
C(15)-C(14)-Rh(1)	113.9(3)	C(26)-C(25)-Rh(2)	68.19(19)
C(16)-C(15)-C(14)	121.4(4)	C(22)-C(26)-C(25)	106.4(3)
C(16)-C(15)-C(20)	124.5(5)	C(22)-C(26)-C(32)	126.2(4)
C(14)-C(15)-C(20)	114.2(4)	C(25)-C(26)-C(32)	126.2(4)
C(17)-C(16)-C(15)	119.5(5)	C(22)-C(26)-Rh(2)	70.9(2)
C(16)-C(17)-C(18)	120.1(4)	C(25)-C(26)-Rh(2)	73.0(2)
C(19)-C(18)-C(17)	120.3(5)	C(32)-C(26)-Rh(2)	130.7(3)
C(18)-C(19)-C(14)	122.0(5)	C(23)-C(27)-C(30)	111.8(3)
N(1)-C(20)-C(15)	116.2(4)	C(23)-C(27)-C(29)	111.3(3)
C(23)-C(22)-C(26)	109.1(4)	C(30)-C(27)-C(29)	108.4(3)
C(23)-C(22)-Rh(2)	72.1(2)	C(23)-C(27)-C(28)	106.4(3)
C(26)-C(22)-Rh(2)	70.6(2)	C(30)-C(27)-C(28)	109.0(3)
C(22)-C(23)-C(24)	107.7(3)	C(29)-C(27)-C(28)	109.9(4)
C(22)-C(23)-C(27)	124.7(4)	C(33)-C(32)-C(34)	109.8(4)

C(33)-C(32)-C(26)	111.5(3)	C(35)-Rh(2)-C(26)	109.11(14)
C(34)-C(32)-C(26)	109.7(3)	N(2)-Rh(2)-C(26)	108.06(13)
C(33)-C(32)-C(31)	109.9(4)	C(35)-Rh(2)-C(22)	97.28(15)
C(34)-C(32)-C(31)	108.9(3)	N(2)-Rh(2)-C(22)	143.41(13)
C(26)-C(32)-C(31)	106.9(3)	C(26)-Rh(2)-C(22)	38.51(14)
C(36)-C(35)-C(40)	116.9(3)	C(35)-Rh(2)-C(23)	119.23(15)
C(36)-C(35)-Rh(2)	129.1(3)	N(2)-Rh(2)-C(23)	161.69(14)
C(40)-C(35)-Rh(2)	113.7(3)	C(26)-Rh(2)-C(23)	64.10(14)
C(37)-C(36)-C(35)	120.8(4)	C(22)-Rh(2)-C(23)	37.72(14)
C(36)-C(37)-C(38)	121.5(4)	C(35)-Rh(2)-C(25)	146.55(14)
C(39)-C(38)-C(37)	119.1(4)	N(2)-Rh(2)-C(25)	100.38(14)
C(38)-C(39)-C(40)	119.9(4)	C(26)-Rh(2)-C(25)	38.81(14)
C(39)-C(40)-C(35)	121.8(4)	C(22)-Rh(2)-C(25)	63.48(15)
C(39)-C(40)-C(41)	123.9(4)	C(23)-Rh(2)-C(25)	62.80(14)
C(35)-C(40)-C(41)	114.2(4)	C(35)-Rh(2)-C(24)	157.03(16)
N(2)-C(41)-C(40)	117.4(4)	N(2)-Rh(2)-C(24)	124.03(14)
C(20)-N(1)-C(21)	120.4(4)	C(26)-Rh(2)-C(24)	63.20(14)
C(20)-N(1)-Rh(1)	116.9(3)	C(22)-Rh(2)-C(24)	62.78(14)
C(21)-N(1)-Rh(1)	122.5(3)	C(23)-Rh(2)-C(24)	37.89(15)
C(41)-N(2)-C(42)	121.1(3)	C(25)-Rh(2)-C(24)	36.25(14)
C(41)-N(2)-Rh(2)	115.7(3)	C(35)-Rh(2)-Cl(2)	86.10(10)
C(42)-N(2)-Rh(2)	122.8(3)	N(2)-Rh(2)-Cl(2)	85.32(8)
C(35)-Rh(2)-N(2)	78.65(15)	C(26)-Rh(2)-Cl(2)	161.16(11)

C(22)-Rh(2)-Cl(2)	131.01(10)
C(23)-Rh(2)-Cl(2)	99.10(9)
C(25)-Rh(2)-Cl(2)	127.31(10)
C(24)-Rh(2)-Cl(2)	98.46(9)
C(14)-Rh(1)-N(1)	78.78(16)
C(14)-Rh(1)-C(5)	103.71(15)
N(1)-Rh(1)-C(5)	109.58(14)
C(14)-Rh(1)-C(4)	97.31(16)
N(1)-Rh(1)-C(4)	146.62(15)
C(5)-Rh(1)-C(4)	38.58(14)
C(14)-Rh(1)-C(3)	123.90(16)
N(1)-Rh(1)-C(3)	156.99(14)
C(5)-Rh(1)-C(3)	64.38(15)
C(4)-Rh(1)-C(3)	37.98(15)
C(14)-Rh(1)-C(1)	139.50(15)
N(1)-Rh(1)-C(1)	98.10(14)
C(5)-Rh(1)-C(1)	38.80(13)
C(4)-Rh(1)-C(1)	63.43(14)
C(3)-Rh(1)-C(1)	62.75(15)
C(14)-Rh(1)-C(2)	160.06(16)
N(1)-Rh(1)-C(2)	119.17(14)
C(5)-Rh(1)-C(2)	63.59(15)
C(4)-Rh(1)-C(2)	63.03(14)
C(3)-Rh(1)-C(2)	37.82(14)
C(1)-Rh(1)-C(2)	36.47(14)
C(14)-Rh(1)-Cl(1)	87.81(12)
N(1)-Rh(1)-Cl(1)	85.69(10)
C(5)-Rh(1)-Cl(1)	162.23(11)
C(4)-Rh(1)-Cl(1)	127.53(11)
C(3)-Rh(1)-Cl(1)	98.00(11)
C(1)-Rh(1)-Cl(1)	132.52(10)
C(2)-Rh(1)-Cl(1)	101.41(11)

Symmetry transformations used to generate equivalent atoms:

Table 4. Anisotropic displacement parameters ($Å^2$x 10^3)for Rovis167_0m. The anisotropic displacement factor exponent takes the form: $-2p^2[h^2a^{*2}U^{11} + ... + 2 h k a^* b^* U^{12}]$

	U^{11}	U^{22}	U^{33}	U^{23}	U^{13}	U^{12}
C(1)	18(2)	24(2)	22(2)	-1(2)	4(2)	-5(2)
C(2)	24(2)	26(2)	26(2)	-1(2)	0(2)	0(2)
C(3)	24(2)	27(2)	25(2)	-5(2)	-5(2)	2(2)
C(4)	25(2)	20(2)	27(2)	-2(2)	-2(2)	5(2)
C(5)	16(2)	24(2)	25(2)	-2(2)	1(2)	0(2)
C(6)	65(4)	28(2)	34(3)	0(2)	-11(2)	-18(2)
C(7)	20(2)	27(2)	24(2)	1(2)	-2(2)	-4(2)
C(8)	40(3)	77(4)	34(3)	-26(3)	1(2)	-27(3)
C(9)	44(3)	33(2)	64(3)	-1(2)	-27(3)	-8(2)
C(10)	41(3)	33(2)	32(3)	-5(2)	-13(2)	4(2)
C(11)	64(4)	36(2)	38(3)	3(2)	-26(3)	-3(2)
C(12)	46(3)	37(2)	42(3)	-5(2)	-19(2)	-7(2)
C(13)	42(3)	47(3)	60(4)	-14(3)	-28(3)	9(2)
C(14)	41(3)	22(2)	20(2)	-3(2)	2(2)	-7(2)
C(15)	36(3)	32(2)	32(3)	-10(2)	6(2)	-13(2)
C(16)	41(3)	53(3)	41(3)	-17(2)	8(2)	-23(2)
C(17)	84(5)	60(3)	35(3)	-14(3)	22(3)	-55(3)
C(18)	96(5)	33(2)	26(3)	-2(2)	9(3)	-31(3)

C(19)	56(3)	27(2)	23(2)	-1(2)	4(2)	-6(2)
C(20)	23(2)	36(2)	37(3)	-8(2)	2(2)	1(2)
C(21)	43(3)	32(2)	35(3)	9(2)	7(2)	10(2)
C(22)	21(2)	19(2)	32(2)	5(2)	-8(2)	-9(2)
C(23)	19(2)	20(2)	27(2)	1(2)	-6(2)	-6(2)
C(24)	21(2)	20(2)	29(2)	2(2)	-4(2)	-9(2)
C(25)	20(2)	24(2)	27(2)	4(2)	1(2)	-9(2)
C(26)	18(2)	19(2)	31(2)	5(2)	-4(2)	-8(2)
C(27)	19(2)	23(2)	24(2)	-2(2)	-7(2)	-1(2)
C(28)	29(3)	39(2)	42(3)	0(2)	-15(2)	-12(2)
C(29)	29(3)	48(3)	23(2)	0(2)	-6(2)	-1(2)
C(30)	31(3)	30(2)	35(3)	0(2)	-14(2)	3(2)
C(31)	39(3)	27(2)	41(3)	13(2)	-1(2)	-5(2)
C(32)	27(2)	21(2)	31(2)	8(2)	-6(2)	-4(2)
C(33)	44(3)	32(2)	45(3)	12(2)	-23(2)	-8(2)
C(34)	35(3)	30(2)	40(3)	5(2)	-7(2)	9(2)
C(35)	20(2)	16(2)	23(2)	4(2)	-4(2)	-3(2)
C(36)	21(2)	20(2)	30(2)	1(2)	-7(2)	-2(2)
C(37)	26(2)	26(2)	28(2)	-2(2)	-1(2)	-1(2)
C(38)	22(2)	26(2)	36(3)	-2(2)	0(2)	-2(2)
C(39)	21(2)	22(2)	38(3)	2(2)	-10(2)	-5(2)
C(40)	21(2)	16(2)	28(2)	3(2)	-8(2)	-2(2)
C(41)	26(2)	18(2)	28(2)	3(2)	-14(2)	-3(2)

C(42)	34(3)	30(2)	25(2)	-3(2)	-5(2)	-8(2)
Cl(2)	25(1)	18(1)	24(1)	4(1)	-6(1)	-5(1)
Cl(1)	39(1)	32(1)	31(1)	-9(1)	0(1)	-2(1)
N(1)	26(2)	26(2)	27(2)	1(1)	3(2)	3(2)
N(2)	28(2)	20(2)	18(2)	2(1)	-7(2)	-6(1)
Rh(2)	17(1)	16(1)	18(1)	2(1)	-5(1)	-4(1)
Rh(1)	24(1)	19(1)	22(1)	1(1)	-1(1)	-1(1)

Table 5. Hydrogen coordinates (x 10^4) and isotropic displacement parameters ($Å^2$x 10^3) for Rovis167_0m.

	x	y	z	U(eq)
H(6A)	8098	5071	2915	61
H(6B)	7018	5200	2358	61
H(6C)	6911	5723	3362	61
H(8A)	9331	5925	1551	72
H(8B)	8756	6958	978	72
H(8C)	8304	5836	977	72
H(9A)	6322	6937	1542	67
H(9B)	6889	7981	1624	67
H(9C)	6135	7512	2529	67
H(11A)	9121	7160	5962	67
H(11B)	10364	6573	6129	67
H(11C)	9642	6054	5477	67
H(12A)	10980	8822	4492	60
H(12B)	11152	8309	5532	60
H(12C)	9925	8895	5322	60
H(13A)	11309	5981	4163	73
H(13B)	12023	6558	4779	73

H(13C)	11789	7053	3746	73
H(16)	3579	7610	5275	53
H(17)	3920	5925	6080	69
H(18)	5809	5062	6147	61
H(19)	7343	5895	5451	44
H(20)	4451	9216	4256	40
H(21A)	5324	10538	3403	60
H(21B)	6430	10057	2710	60
H(21C)	6562	10605	3657	60
H(28A)	10839	2812	935	52
H(28B)	10585	2727	2071	52
H(28C)	9819	3610	1477	52
H(29A)	8060	2869	2337	50
H(29B)	8821	1937	2887	50
H(29C)	7931	1641	2255	50
H(30A)	9563	380	1412	48
H(30B)	10441	754	2013	48
H(30C)	10666	830	876	48
H(31A)	7557	5523	-2213	55
H(31B)	8417	4419	-2318	55
H(31C)	8503	5212	-1516	55
H(33A)	5725	3596	-1234	58
H(33B)	6646	3469	-2165	58

H(33C)	5821	4585	-1995	58
H(34A)	6009	5720	-694	55
H(34B)	7064	5449	-105	55
H(34C)	6043	4762	106	55
H(36)	6045	2738	1986	28
H(37)	4206	2726	2792	33
H(38)	2812	2043	2097	34
H(39)	3261	1432	550	32
H(41)	4827	950	-916	28
H(42A)	6266	521	-2137	44
H(42B)	7495	241	-1798	44
H(42C)	7122	1382	-2318	44

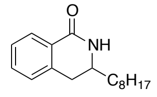

O
NH
C8H17

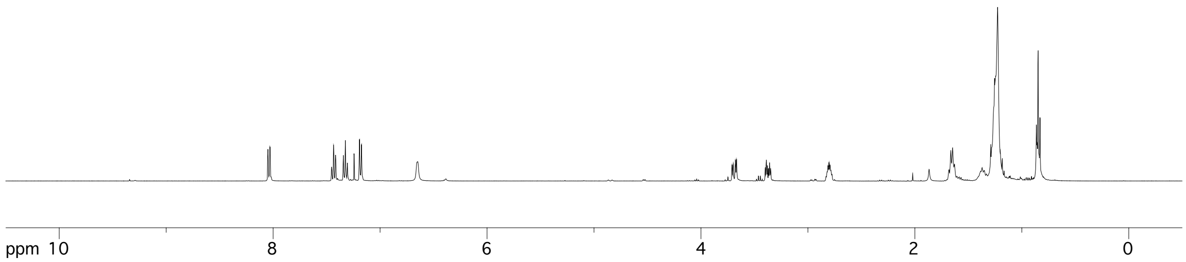

ppm 10
8
6
4
2
0

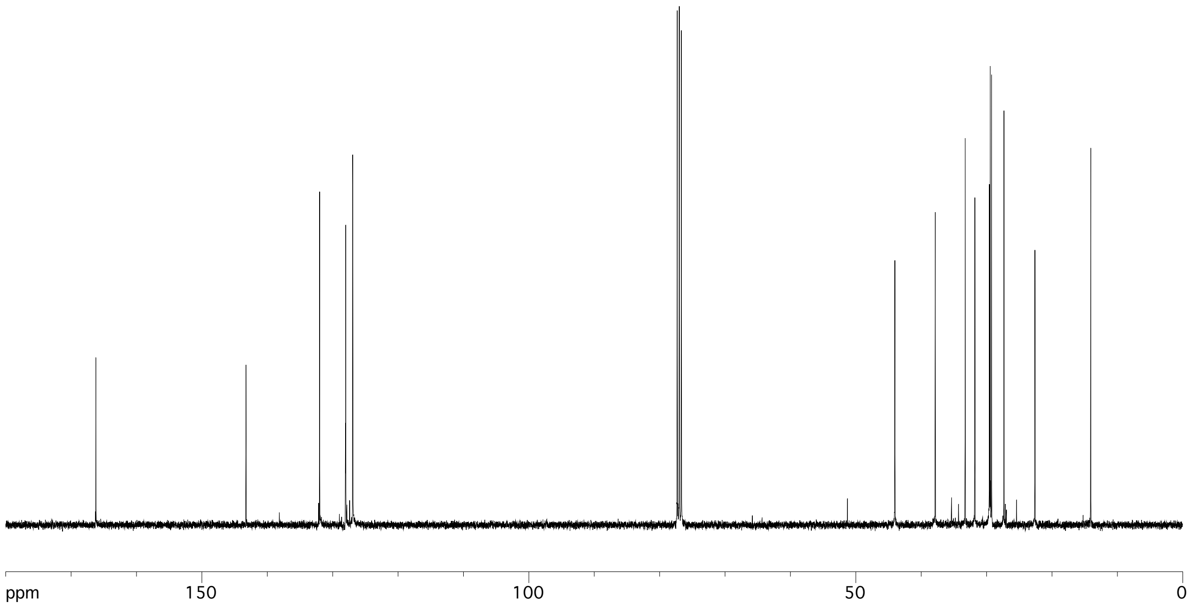

ppm
150
100
50
0

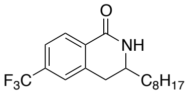
O
NH
F3C
C8H17

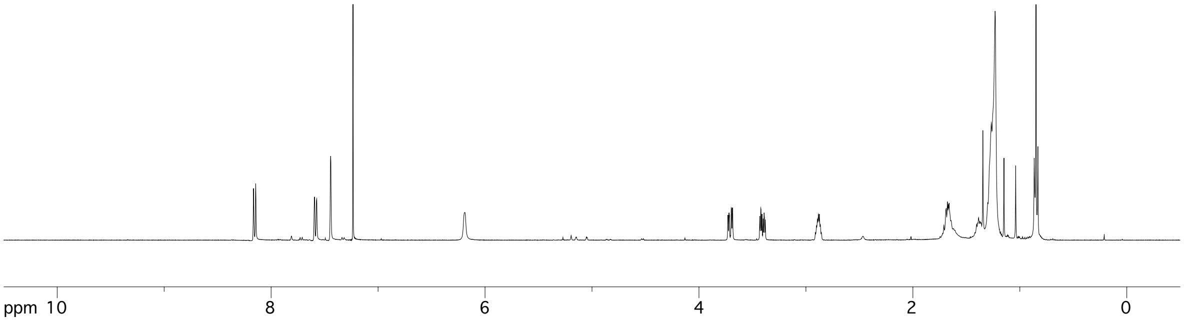
ppm 10
8
6
4
2
0

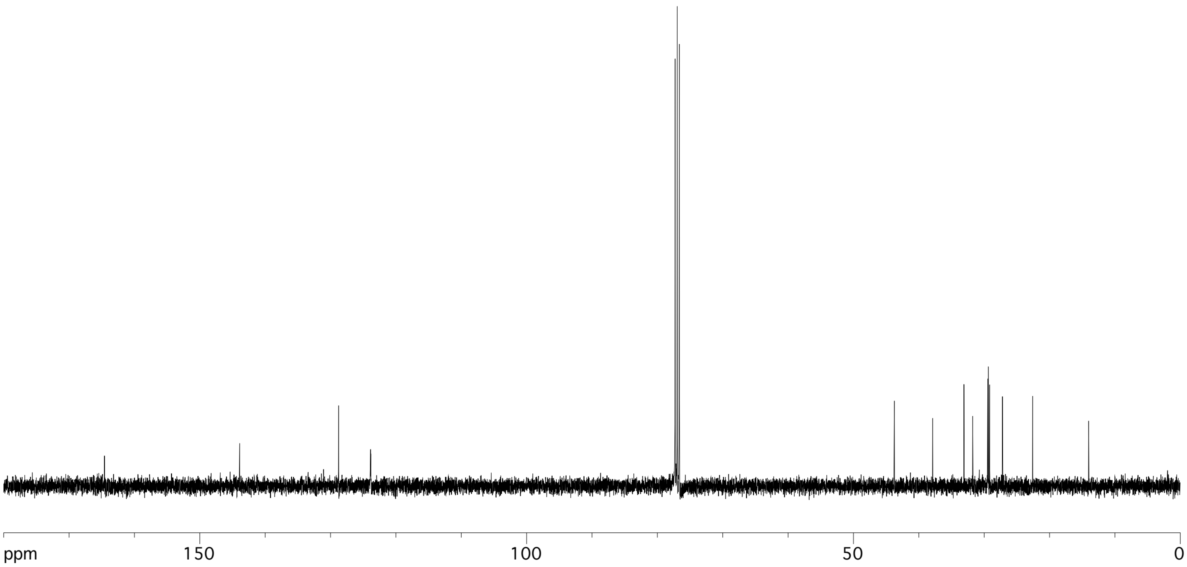
ppm
150
100
50
0

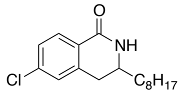
O
NH
Cl
C8H17

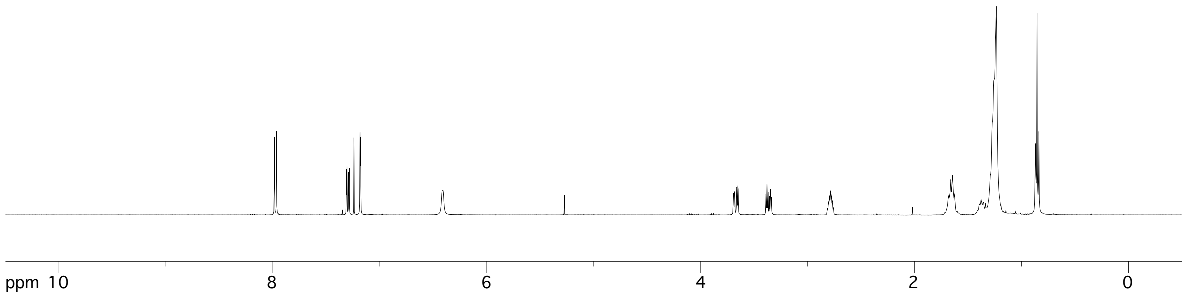
ppm 10
8
6
4
2
0

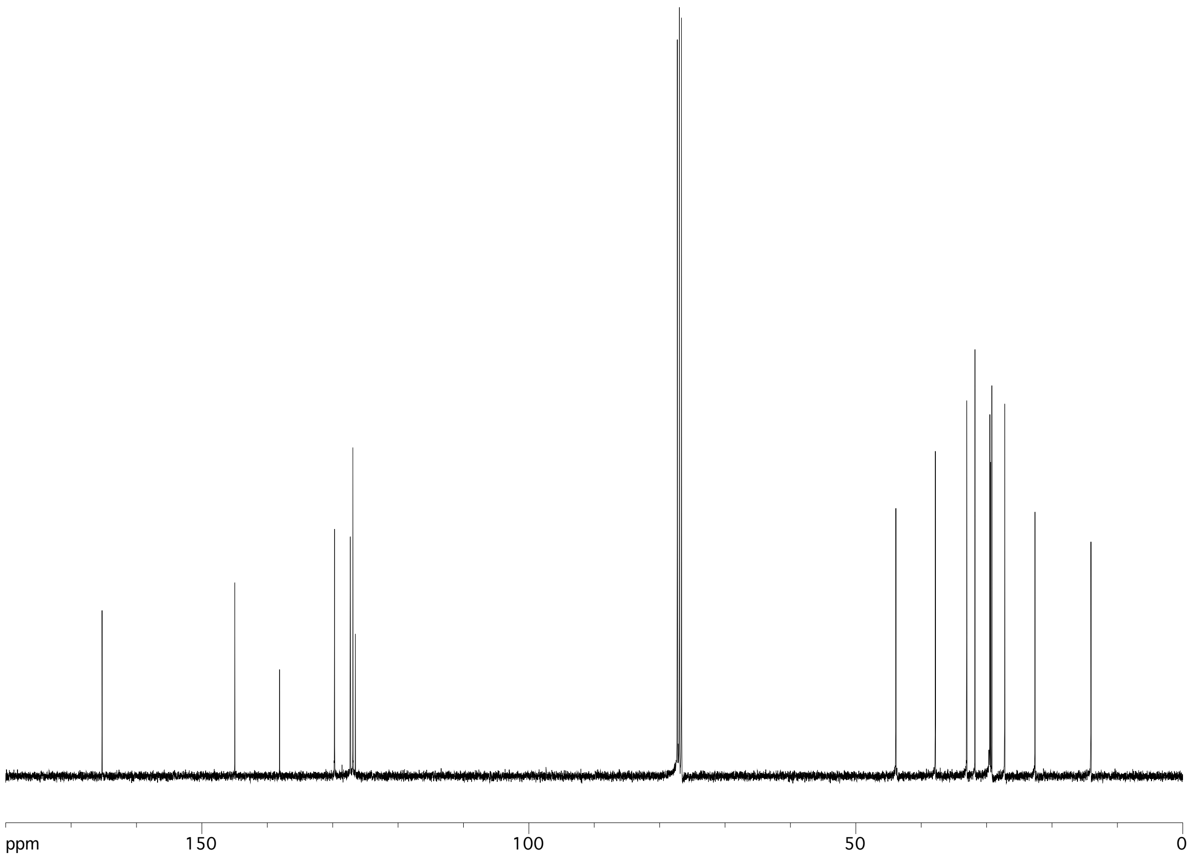
ppm
150
100
50
0

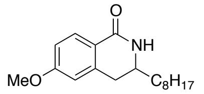
O
NH
MeO
C_8H_{17}

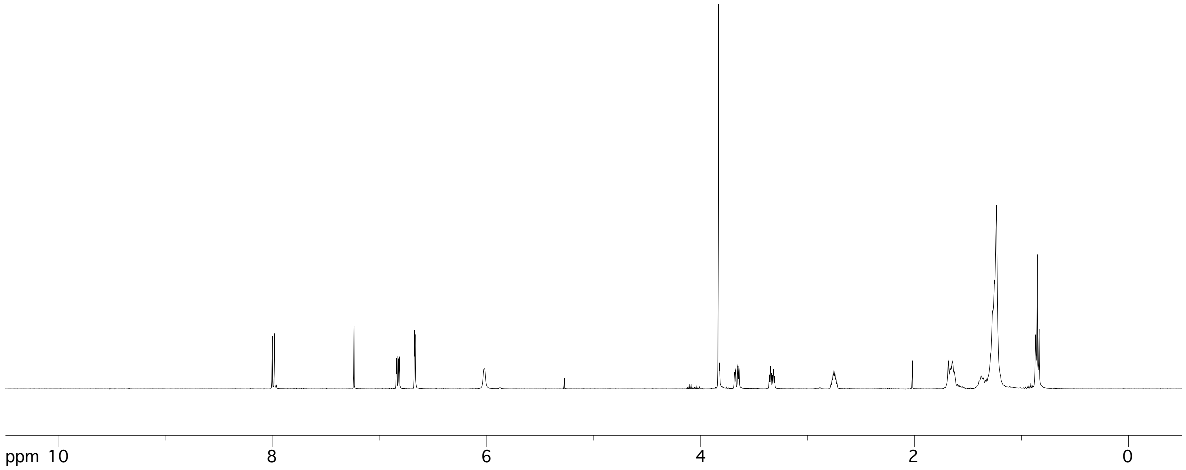
ppm 10
8
6
4
2
0

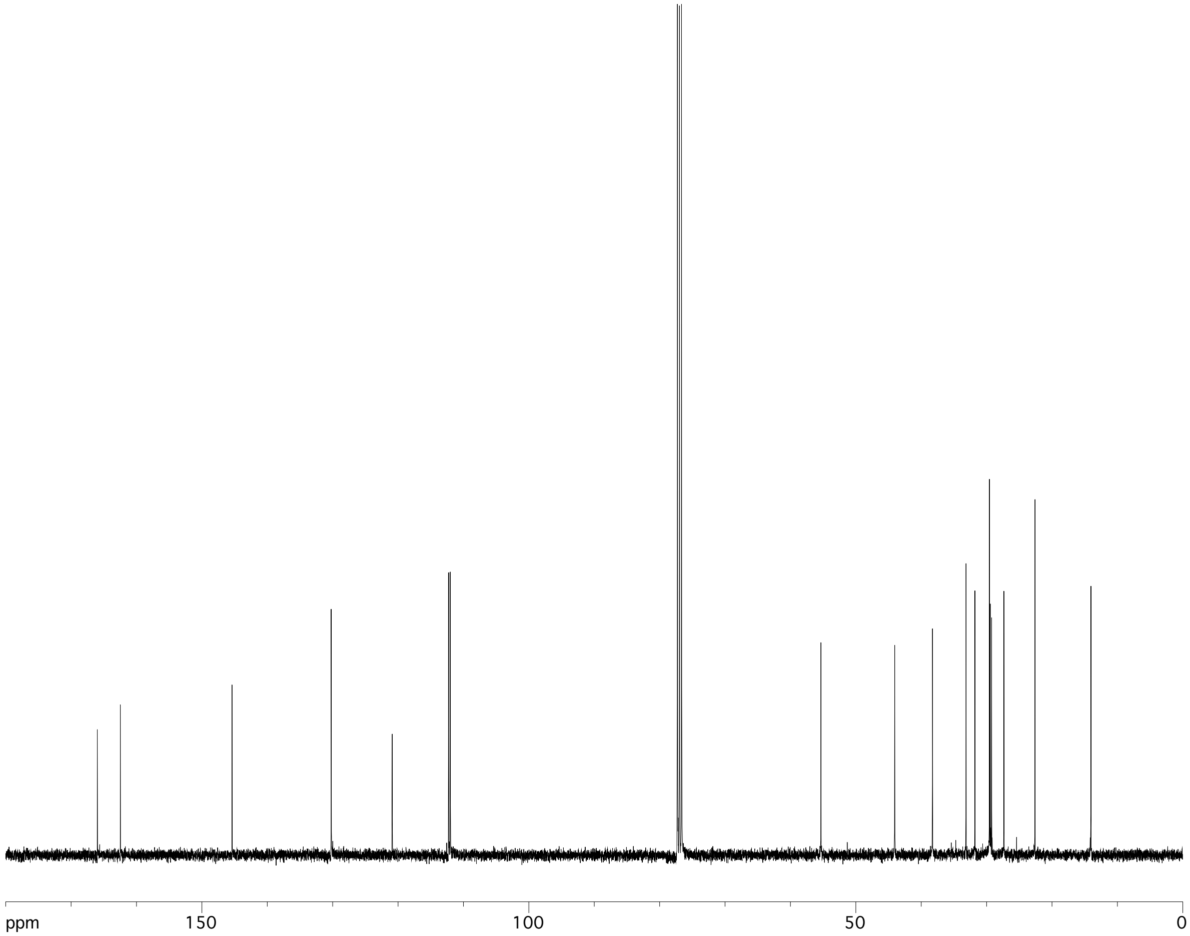
ppm
150
100
50
0

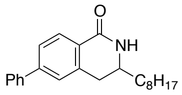
O
NH
Ph
C8H17

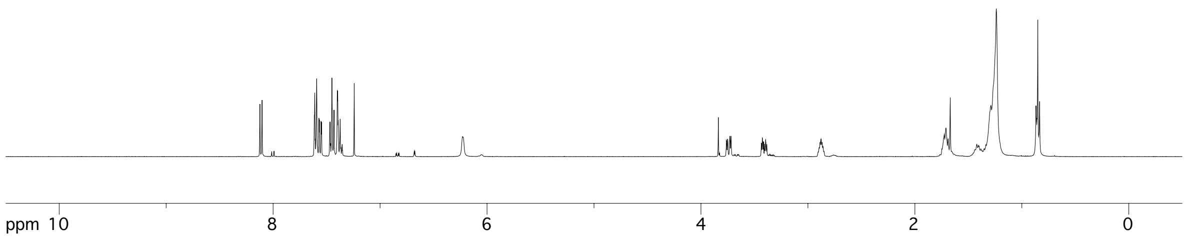
ppm 10
8
6
4
2
0

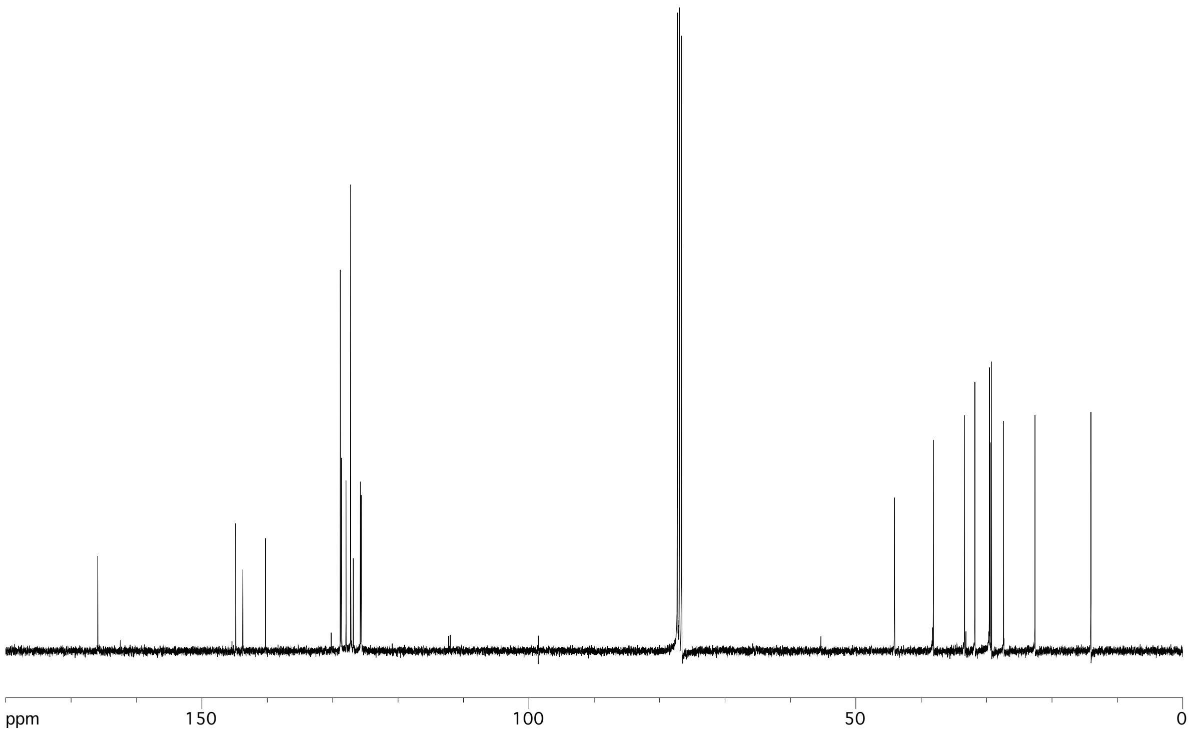
ppm
150
100
50
0

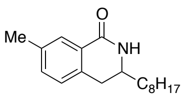
O
Me
NH
C8H17

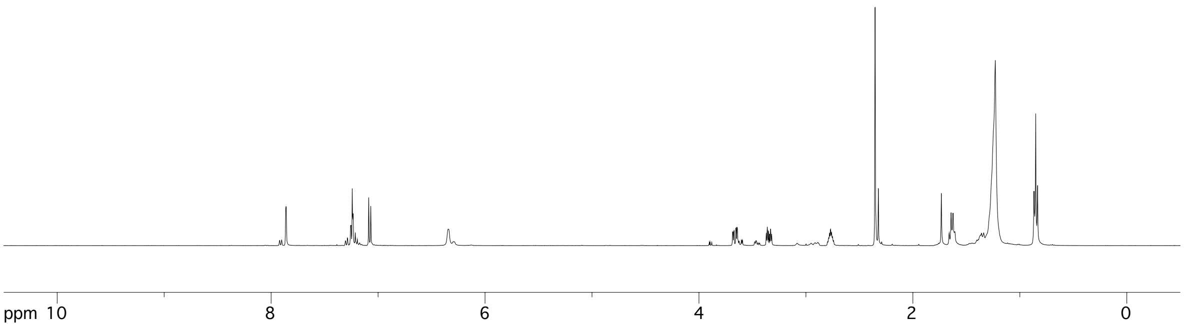
ppm 10
8
6
4
2
0

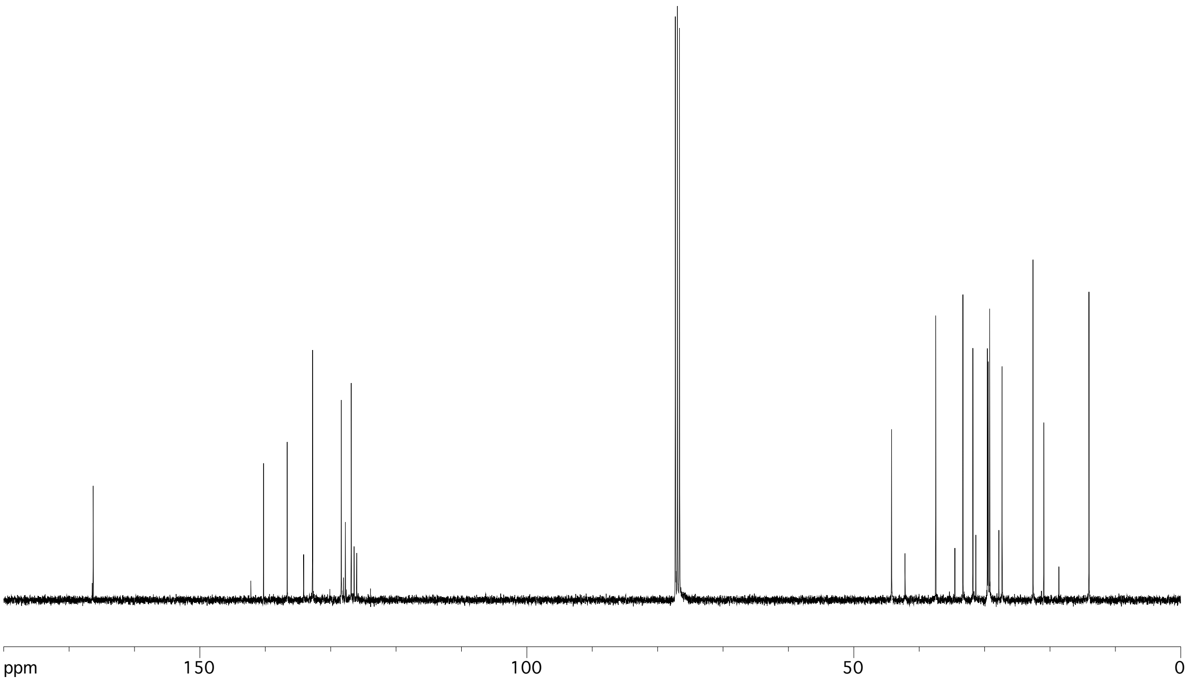
ppm
150
100
50
0

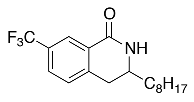
F3C
O
NH
C8H17

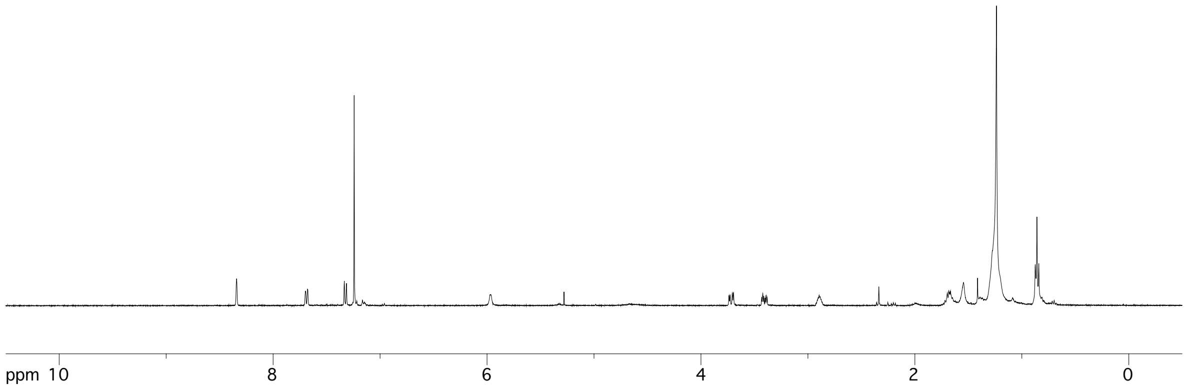
ppm 10
8
6
4
2
0

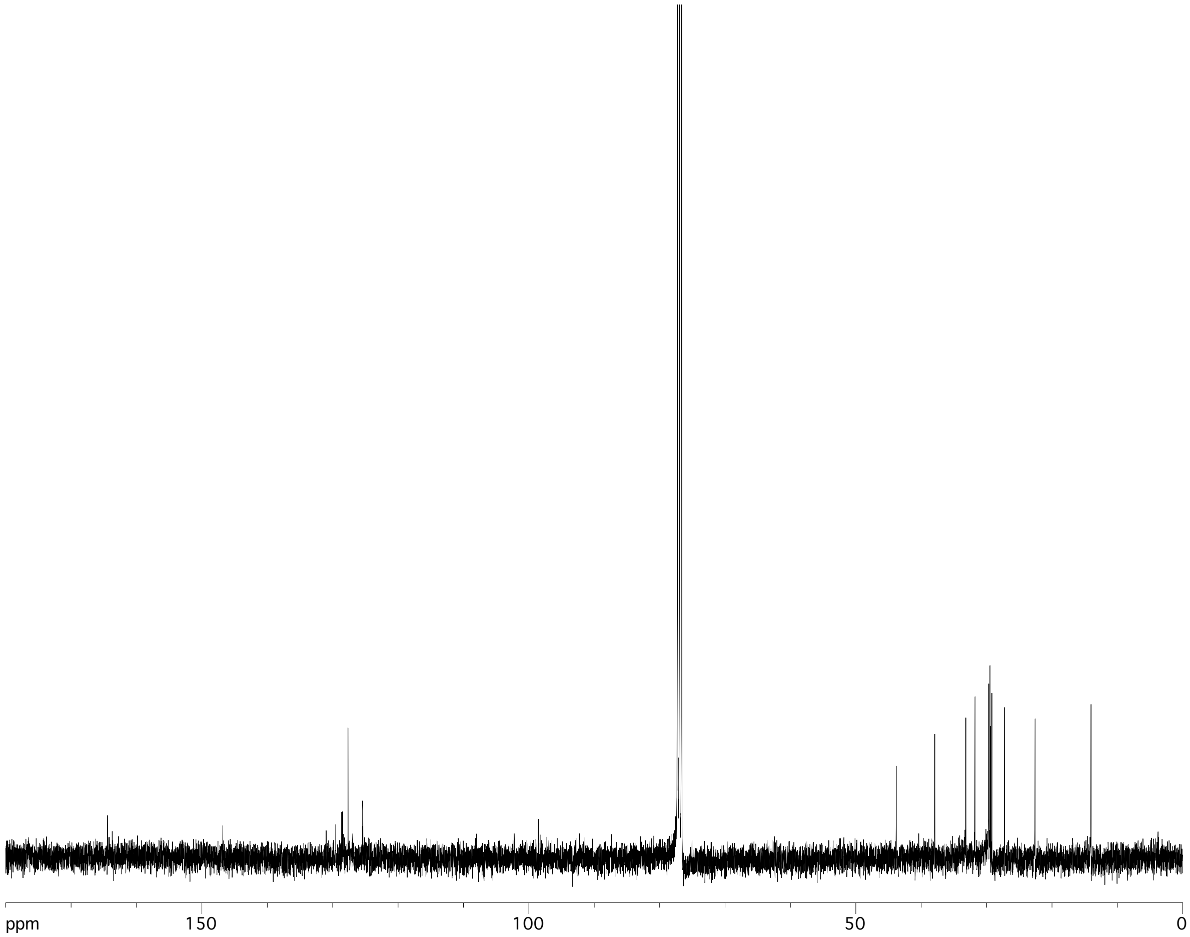
ppm
150
100
50
0

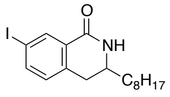
I
O
NH
C8H17

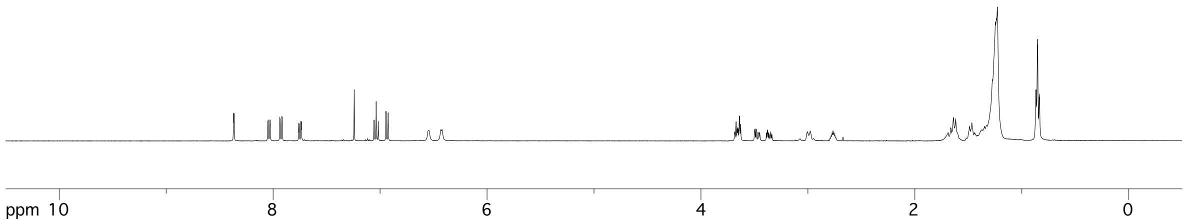
ppm 10
8
6
4
2
0

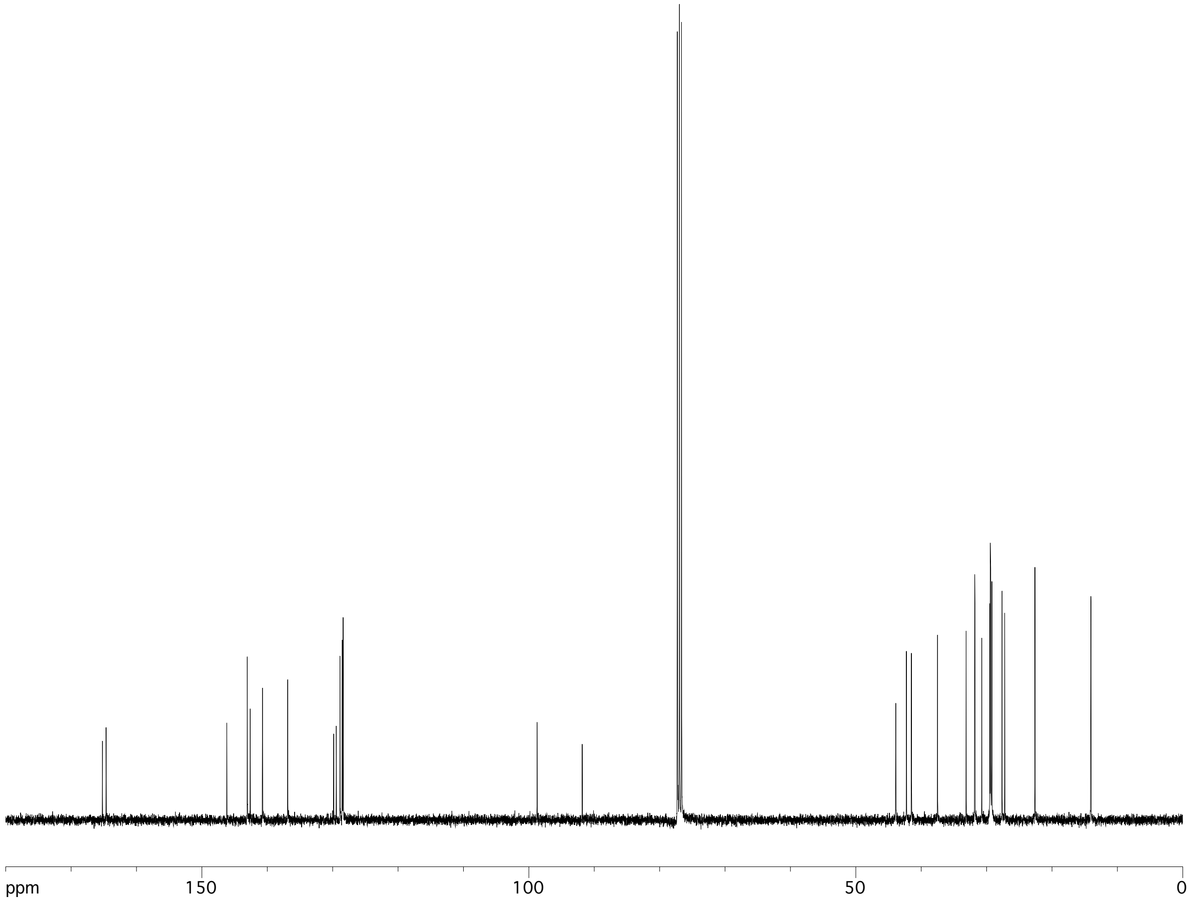
ppm
150
100
50
0

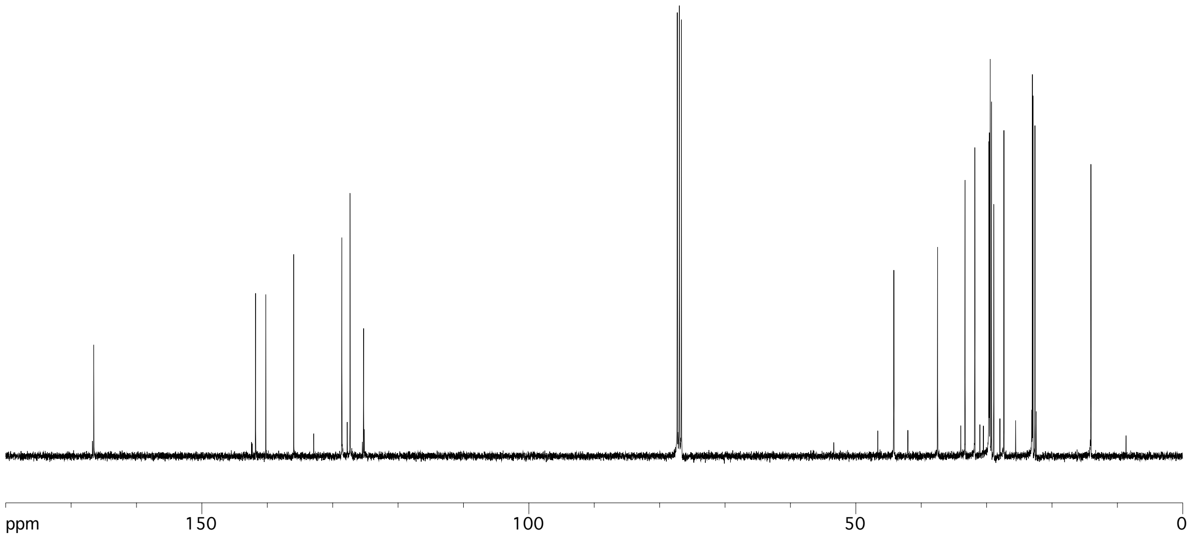
ppm
150
100
50
0

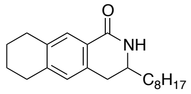
O
NH
C8H17

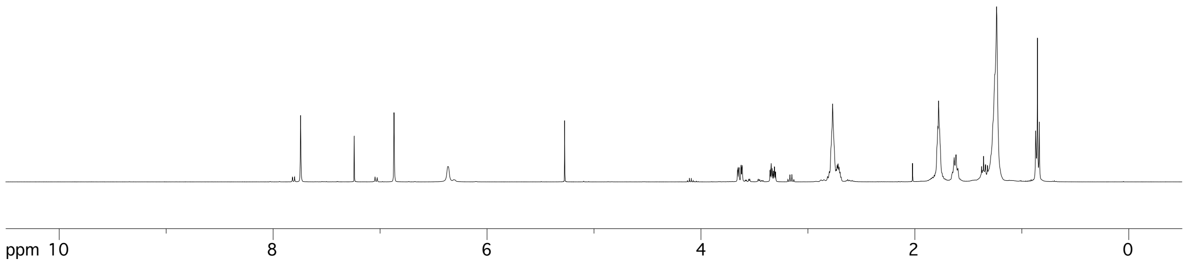
ppm 10
8
6
4
2
0

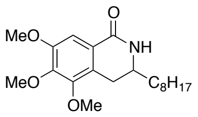
O
MeO
NH
MeO
C8H17
OMe

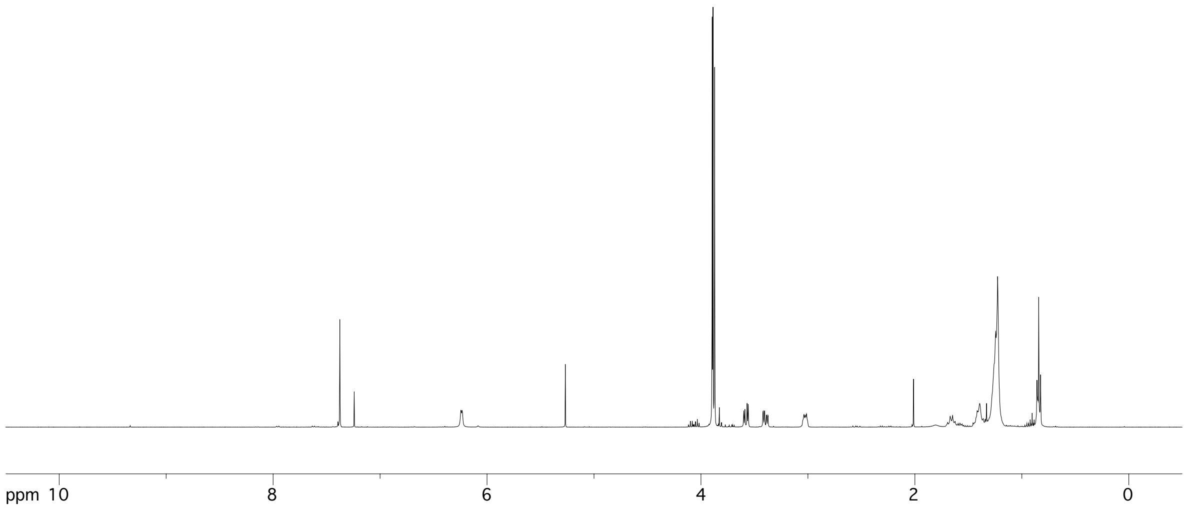
ppm 10
8
6
4
2
0

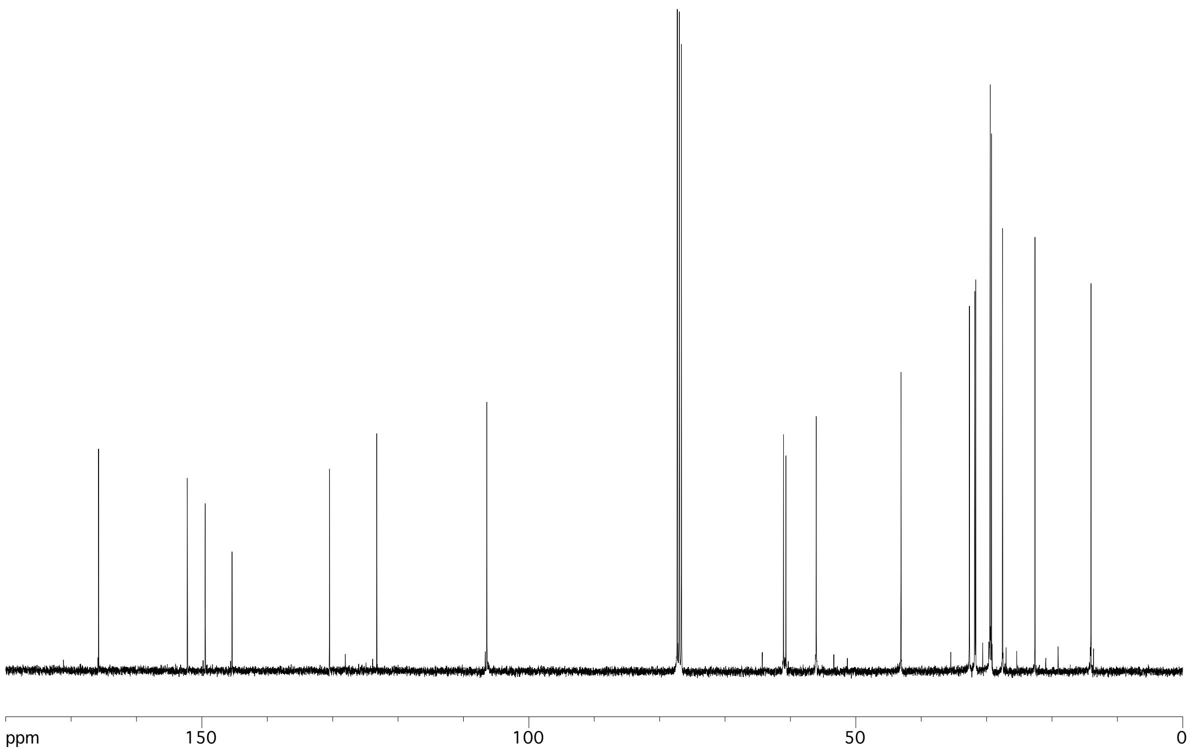
ppm
150
100
50
0

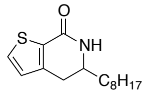
O
S
NH
C_8H_{17}

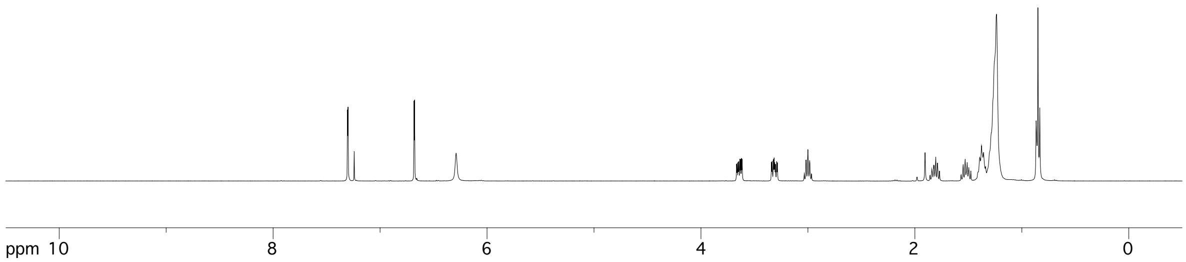
ppm 10
8
6
4
2
0

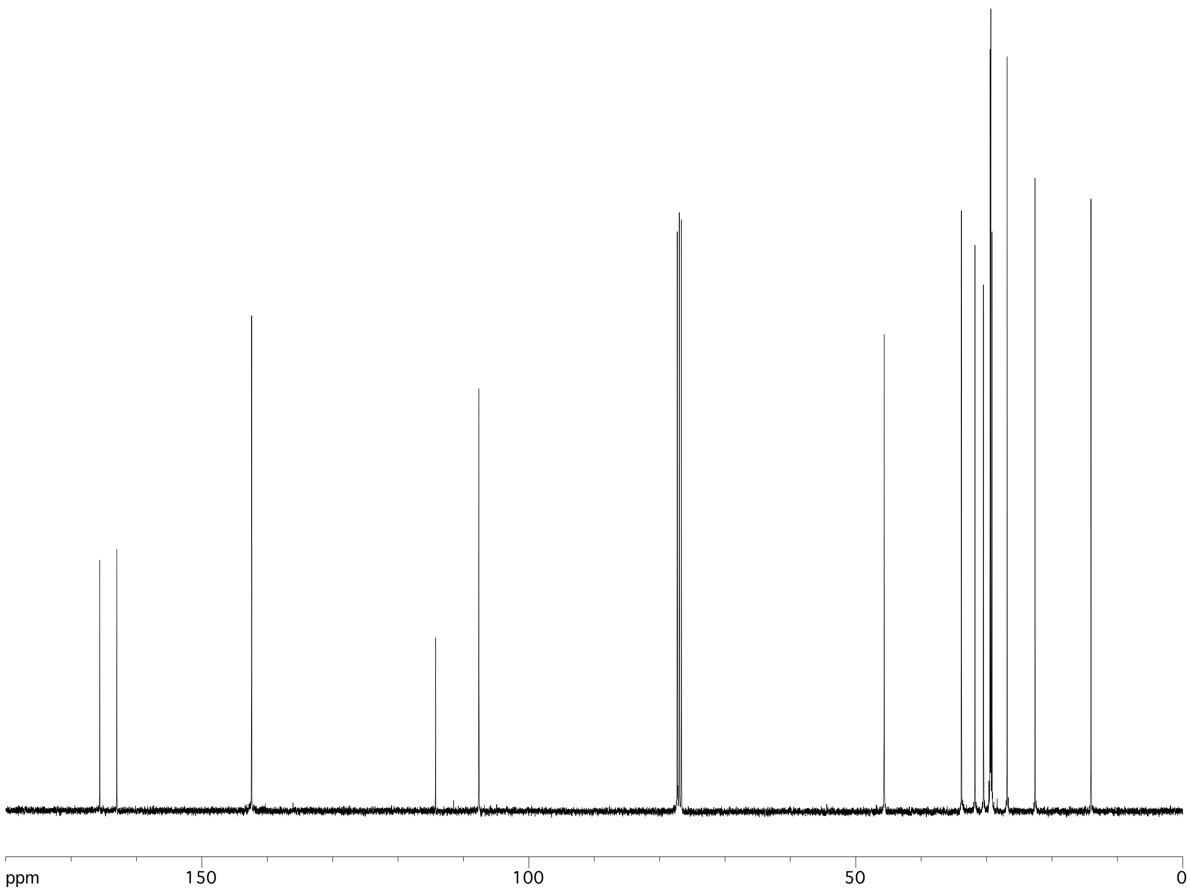
ppm
150
100
50
0

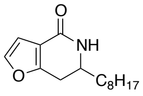
O
NH
O
C_8H_{17}

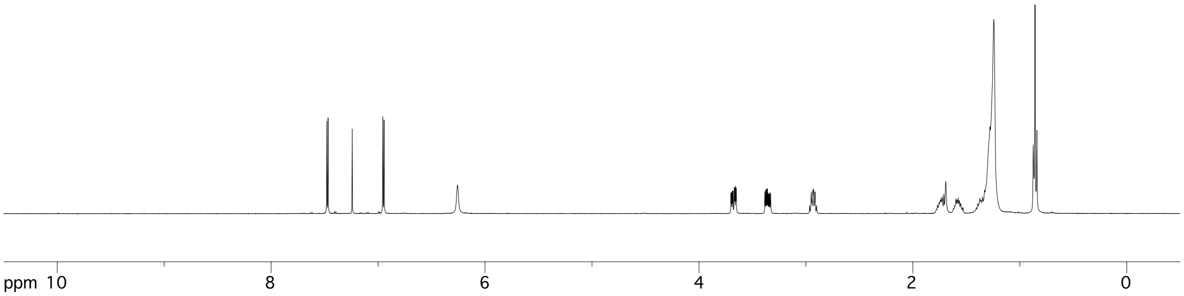
ppm 10
8
6
4
2
0

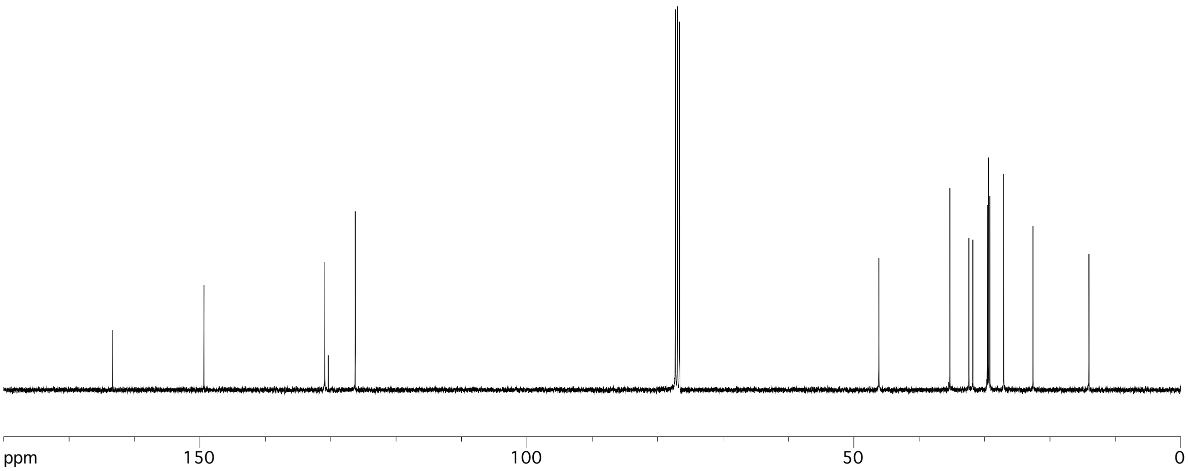
ppm
150
100
50
0

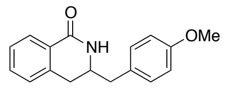
O
NH
OMe

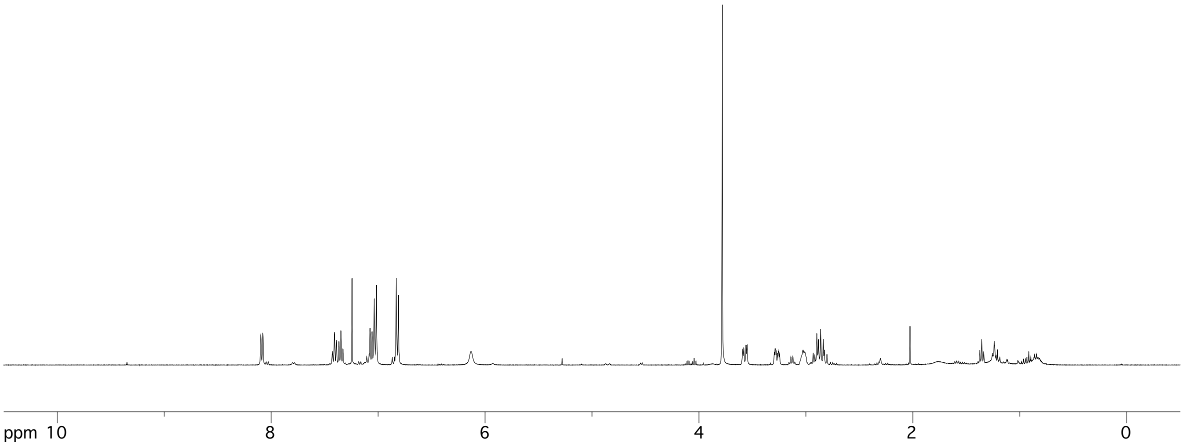
ppm 10
8
6
4
2
0

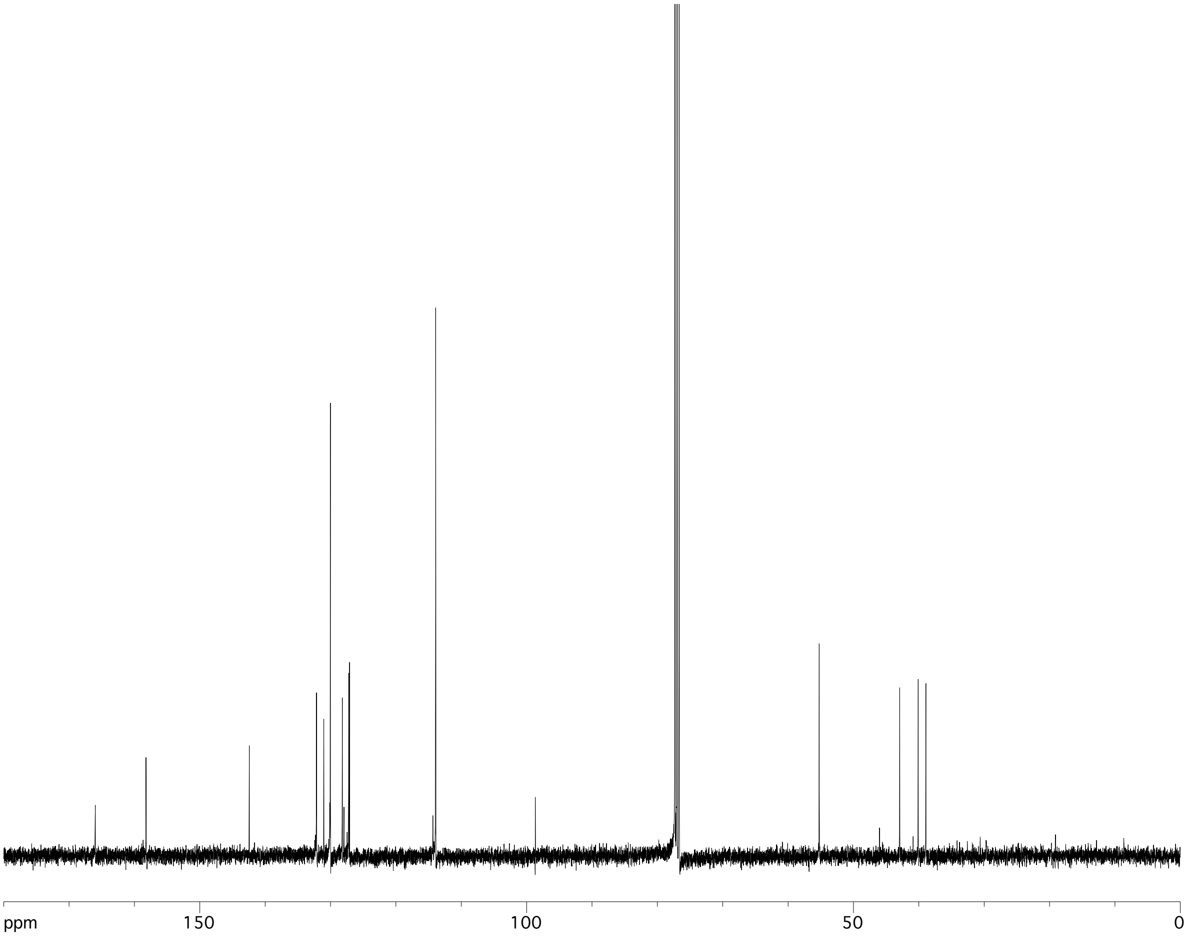
ppm
150
100
50
0

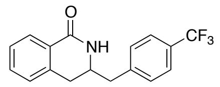
O
NH
CF3

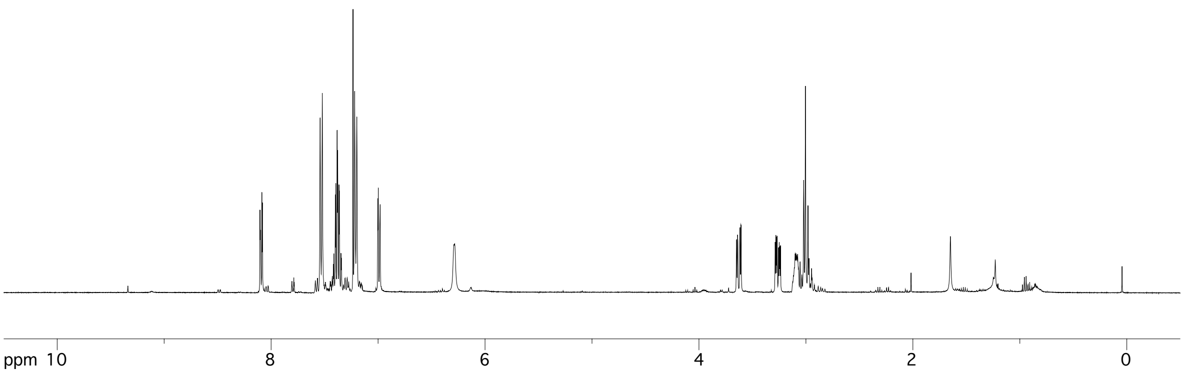
ppm 10
8
6
4
2
0

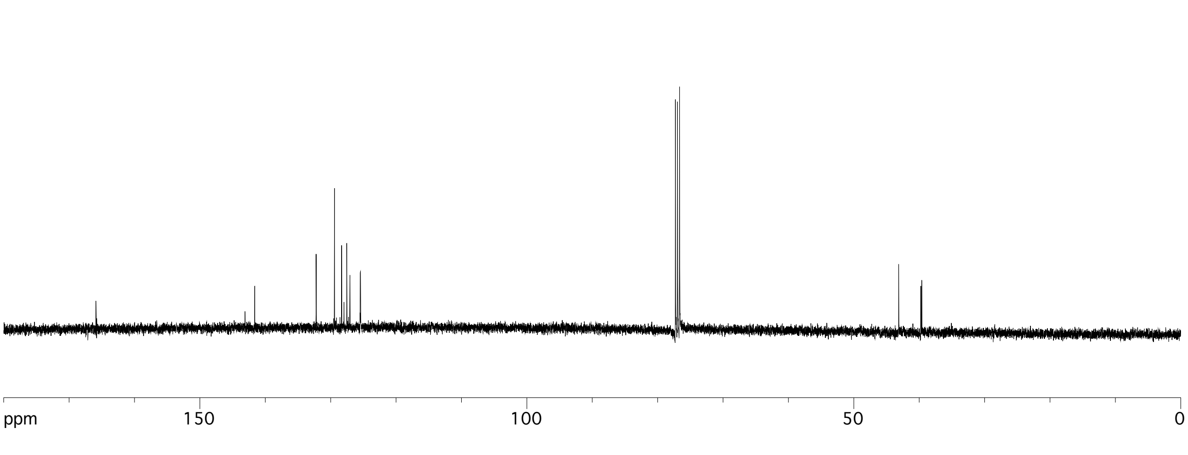
ppm
150
100
50
0

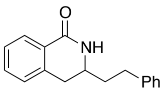
O
NH
Ph

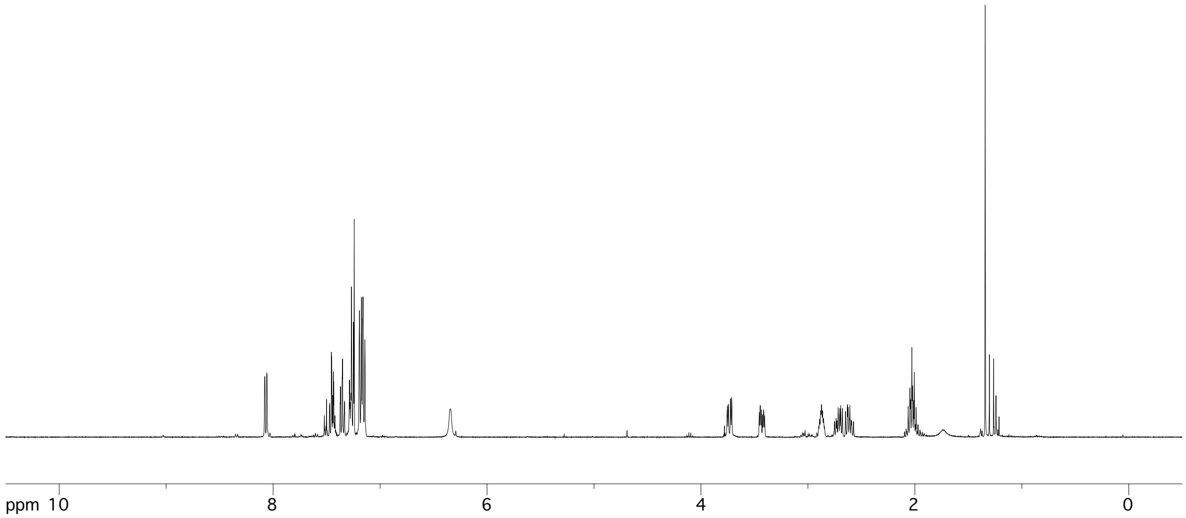
ppm 10
8
6
4
2
0

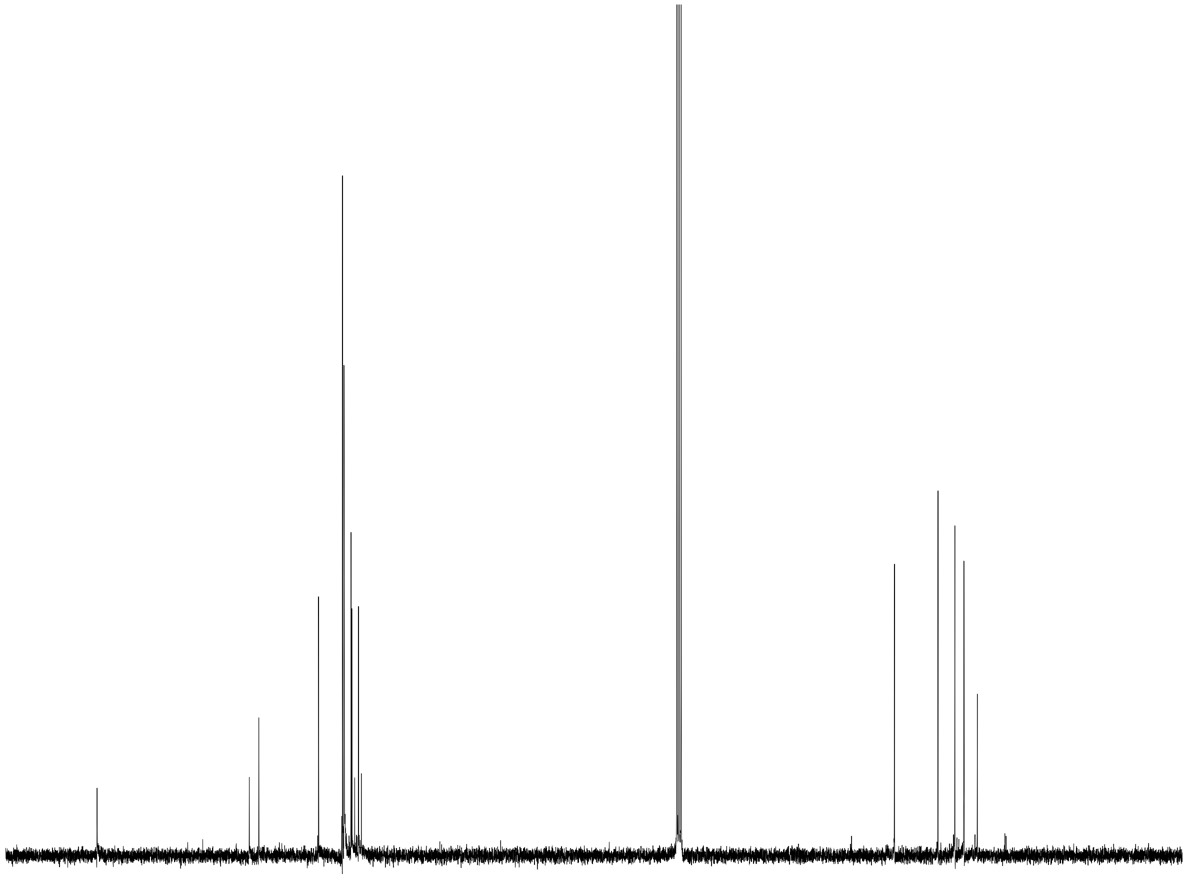
ppm
150
100
50
0

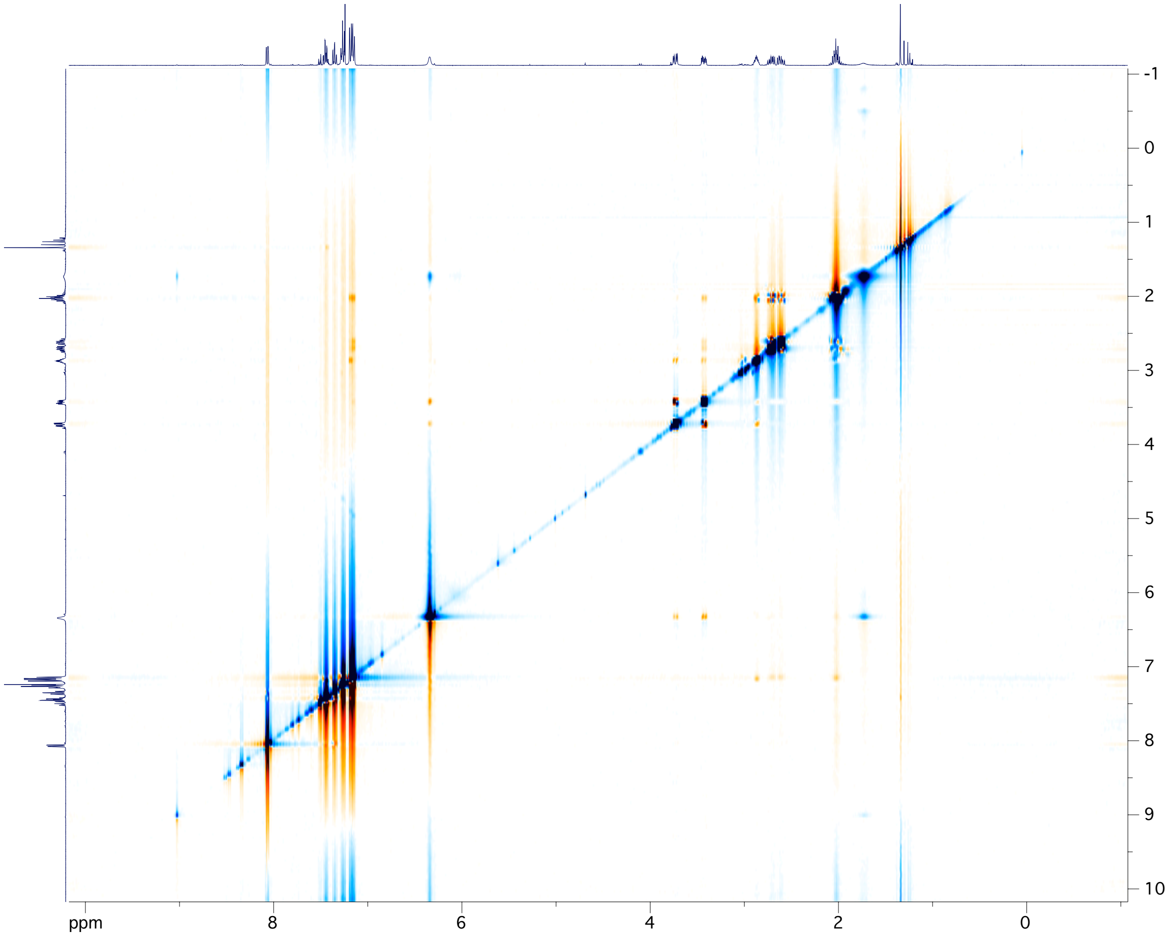

ppm
8
6
4
2
0
-1
0
1
2
3
4
5
6
7
8
9
10

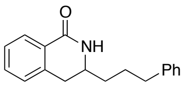
O
NH
Ph

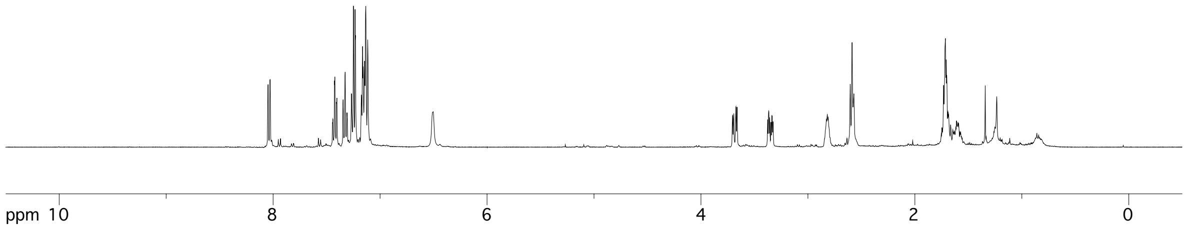
ppm 10
8
6
4
2
0

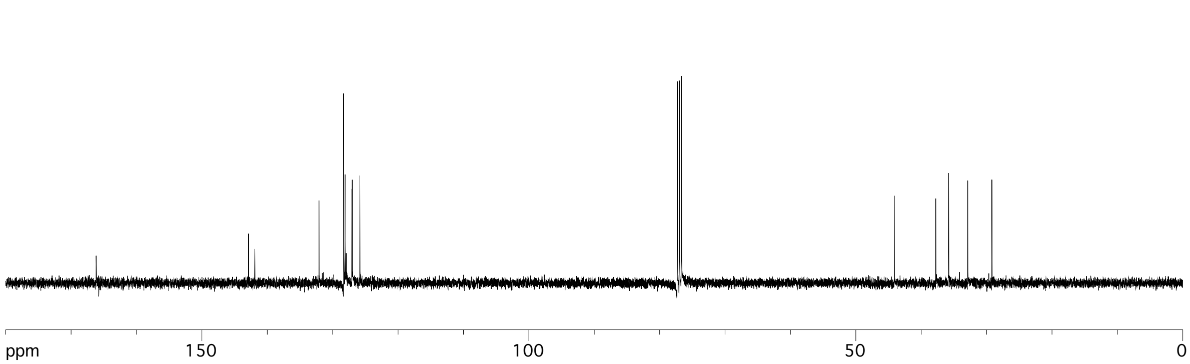
ppm
150
100
50
0

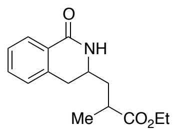
O
NH
Me
CO2Et

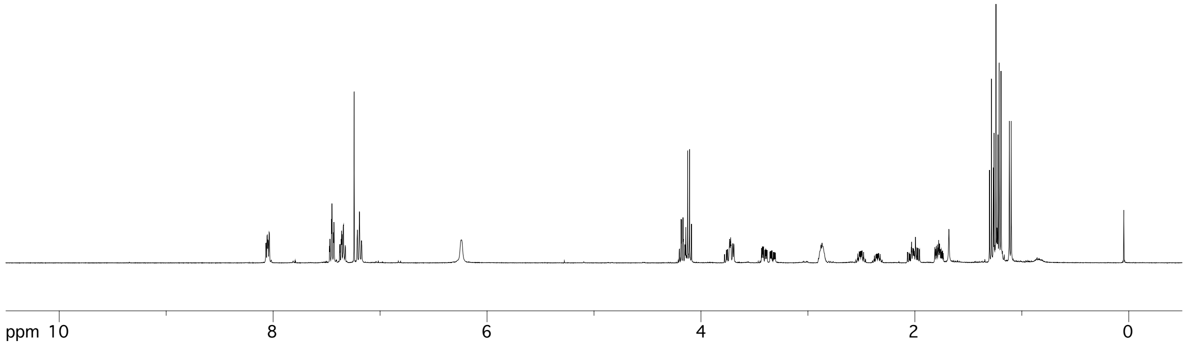
ppm 10
8
6
4
2
0

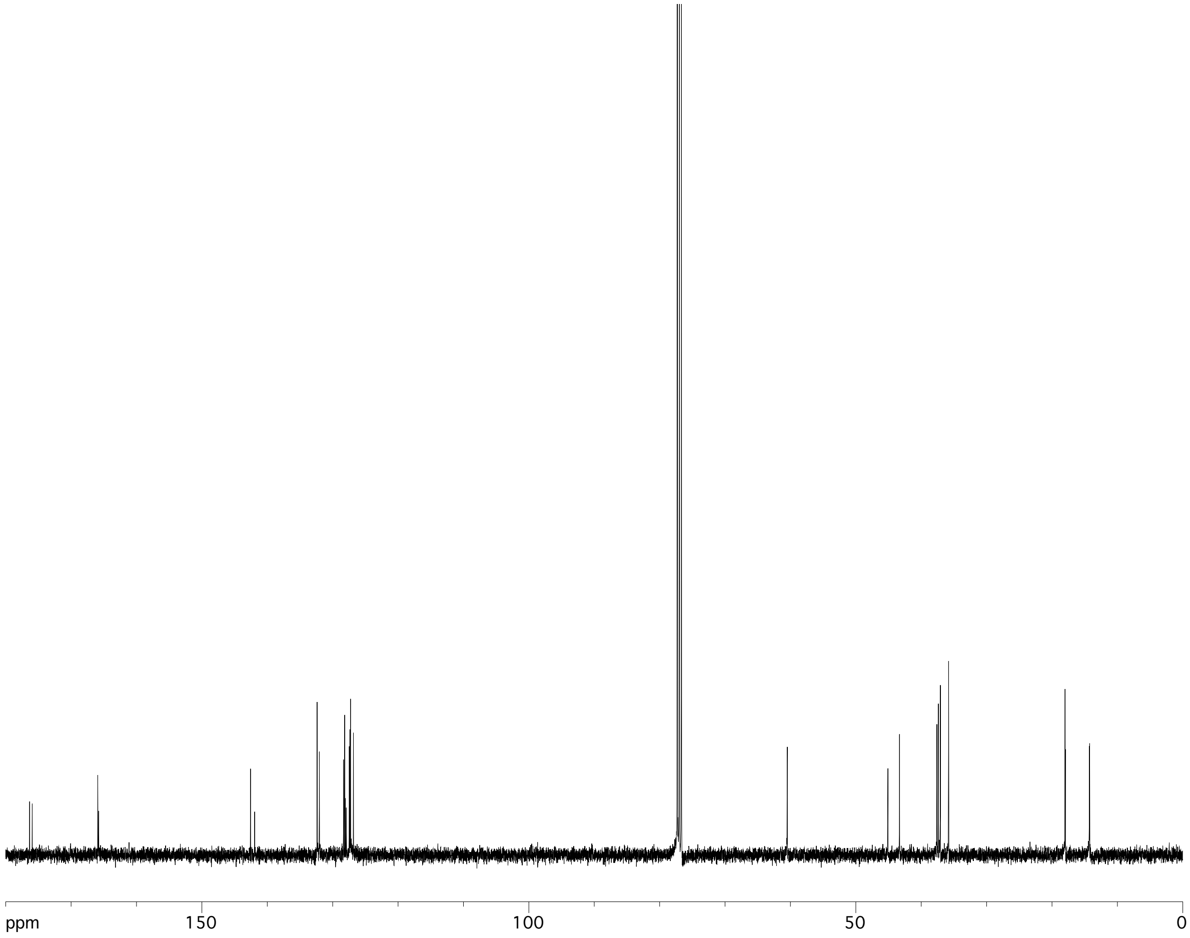
ppm
150
100
50
0

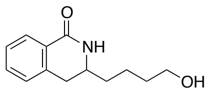
O
NH
OH

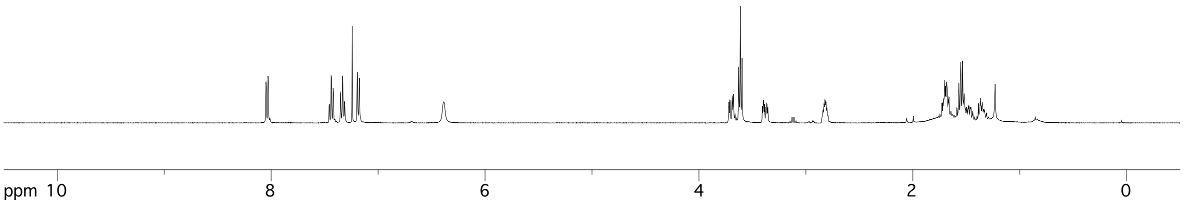
ppm 10
8
6
4
2
0

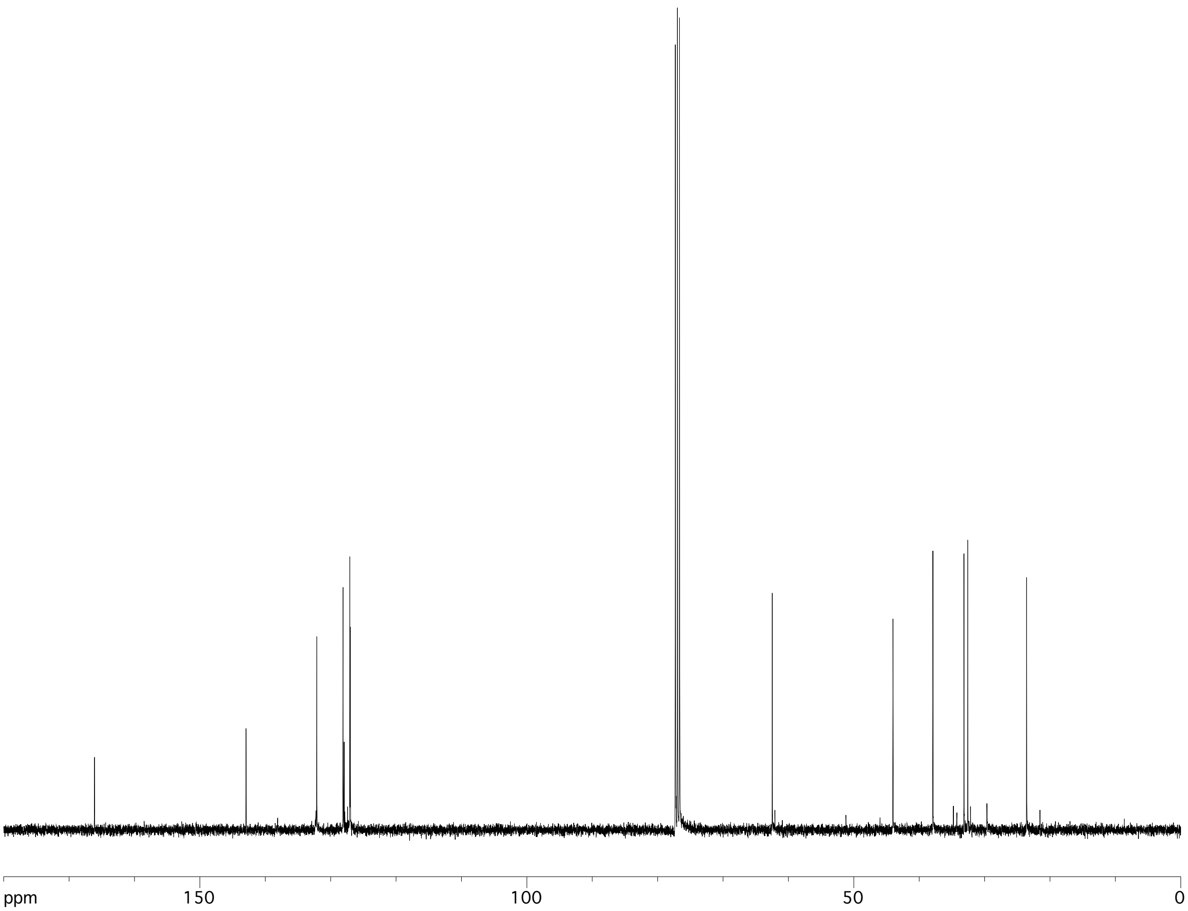
ppm
150
100
50
0

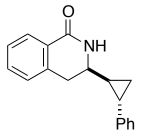
O
NH
Ph

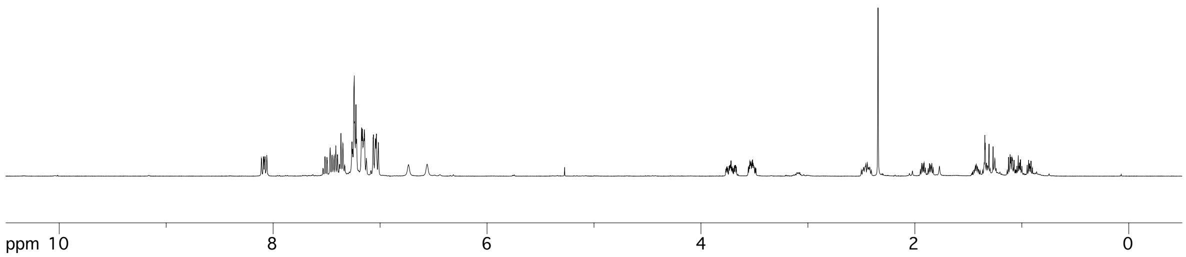
ppm 10
8
6
4
2
0

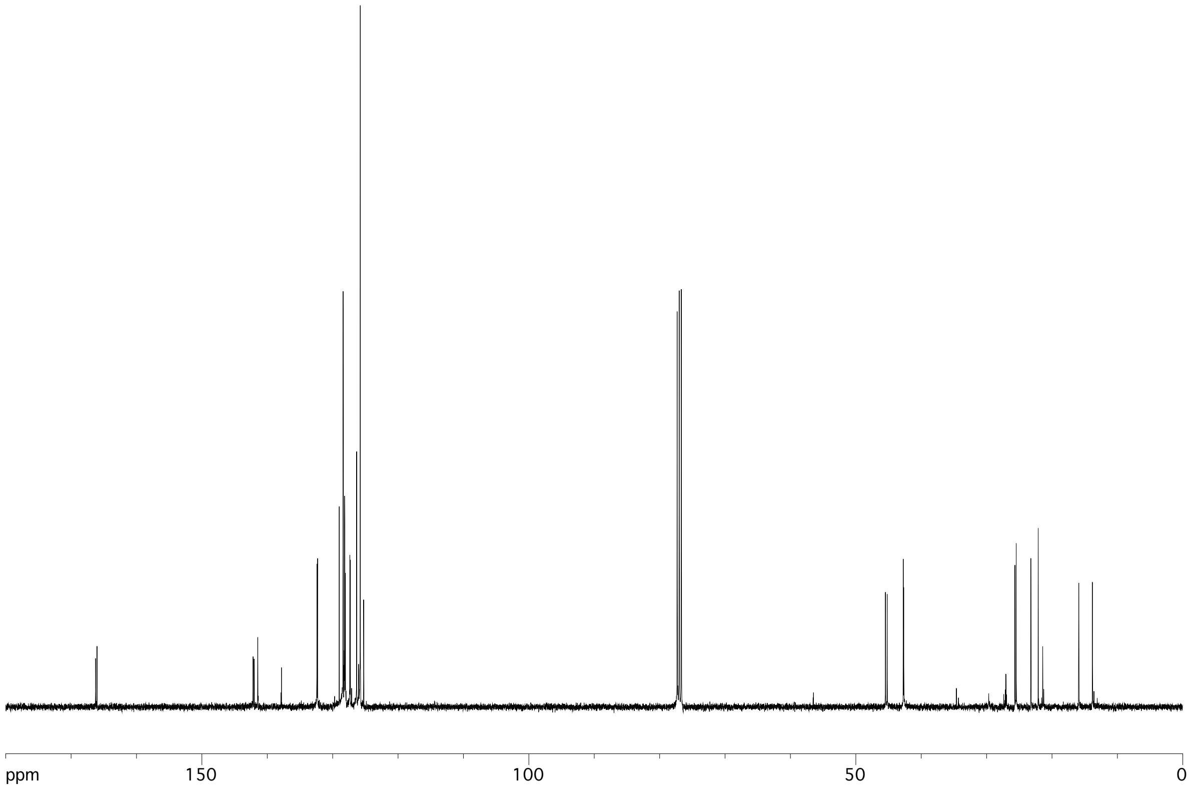
ppm
150
100
50
0

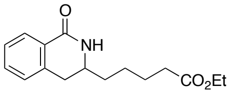
O
NH
CO2Et

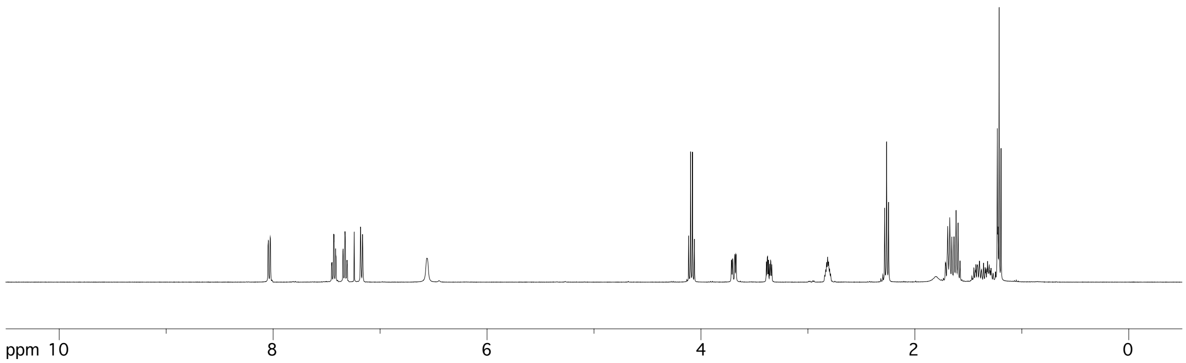
ppm 10
8
6
4
2
0

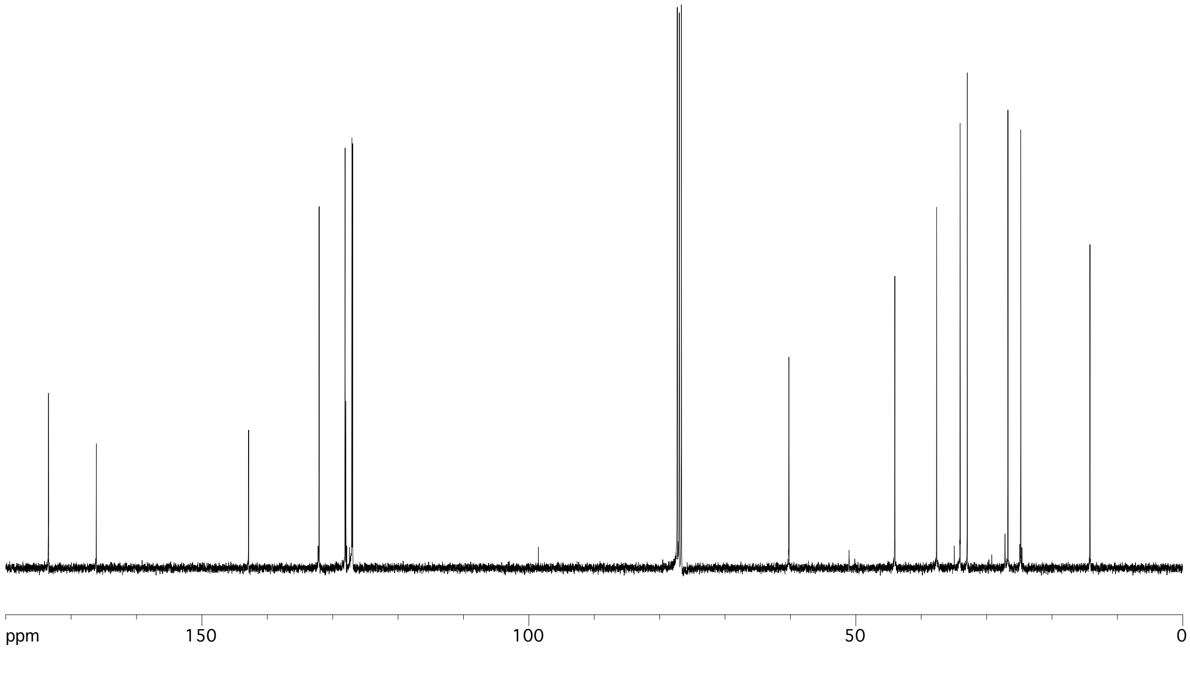
ppm
150
100
50
0

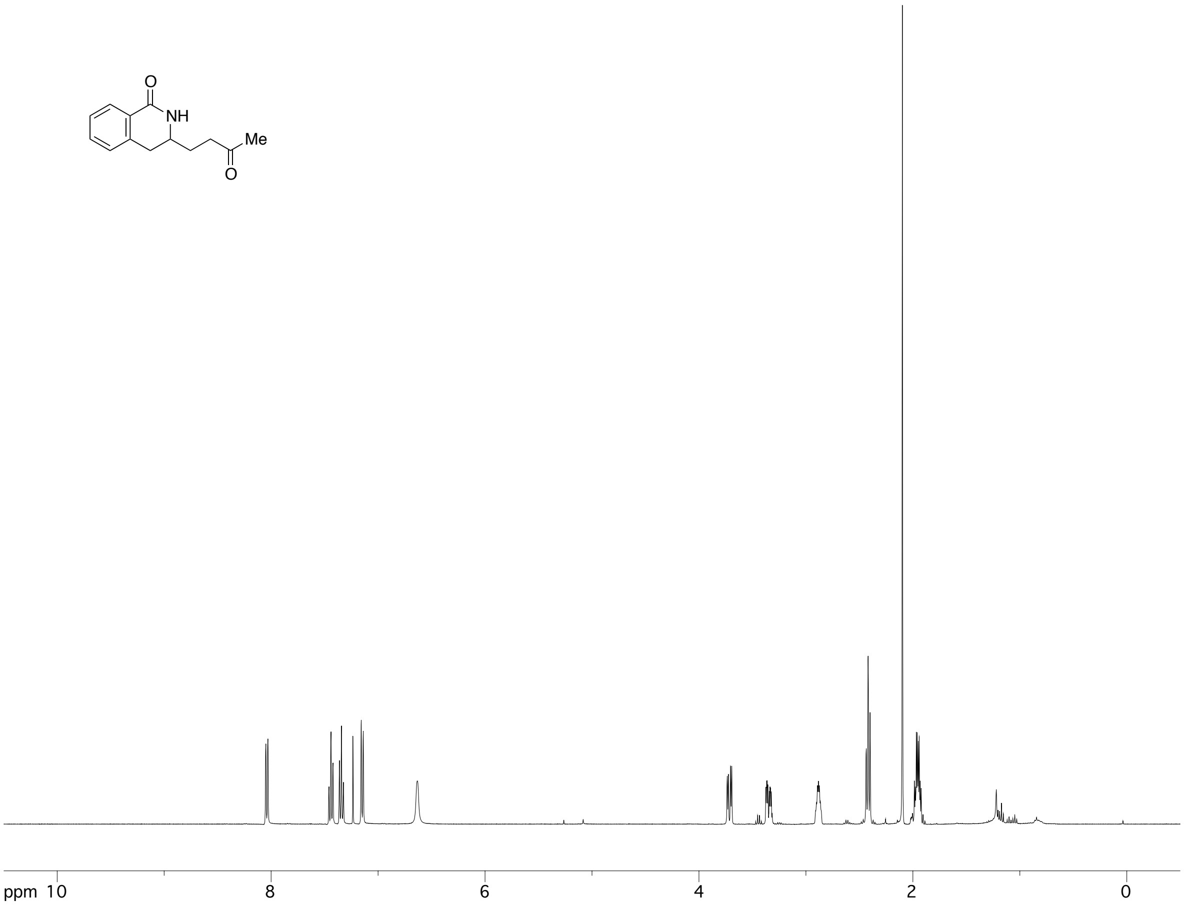
O
NH
Me
O
ppm 10
8
6
4
2
0

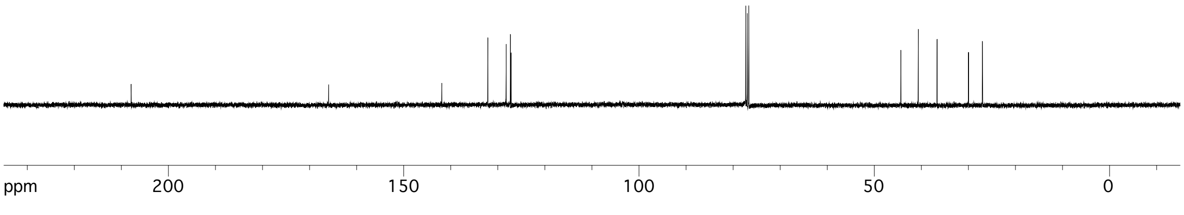
ppm
200
150
100
50
0

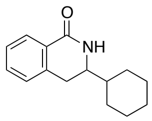
O
NH

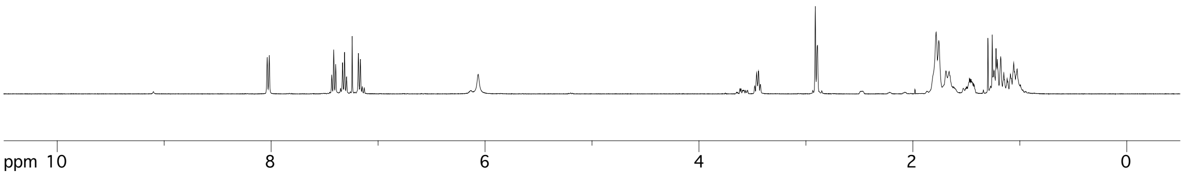
ppm 10
8
6
4
2
0

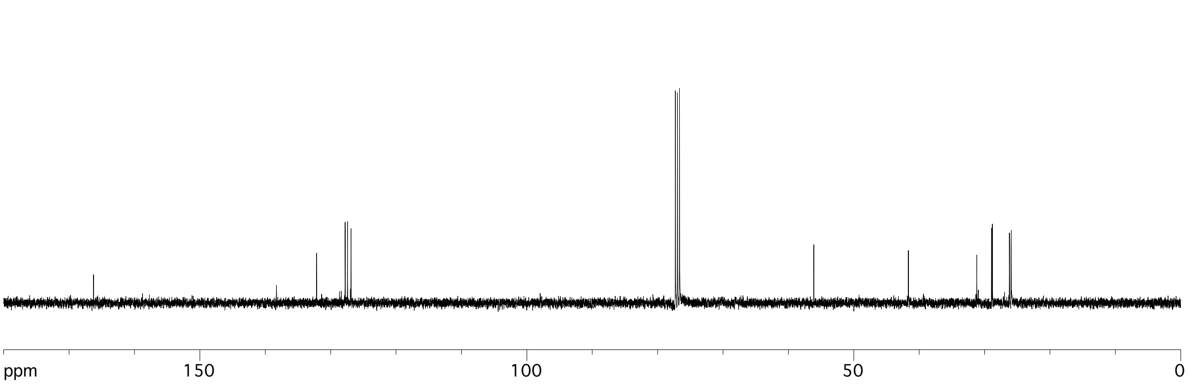
ppm
150
100
50
0

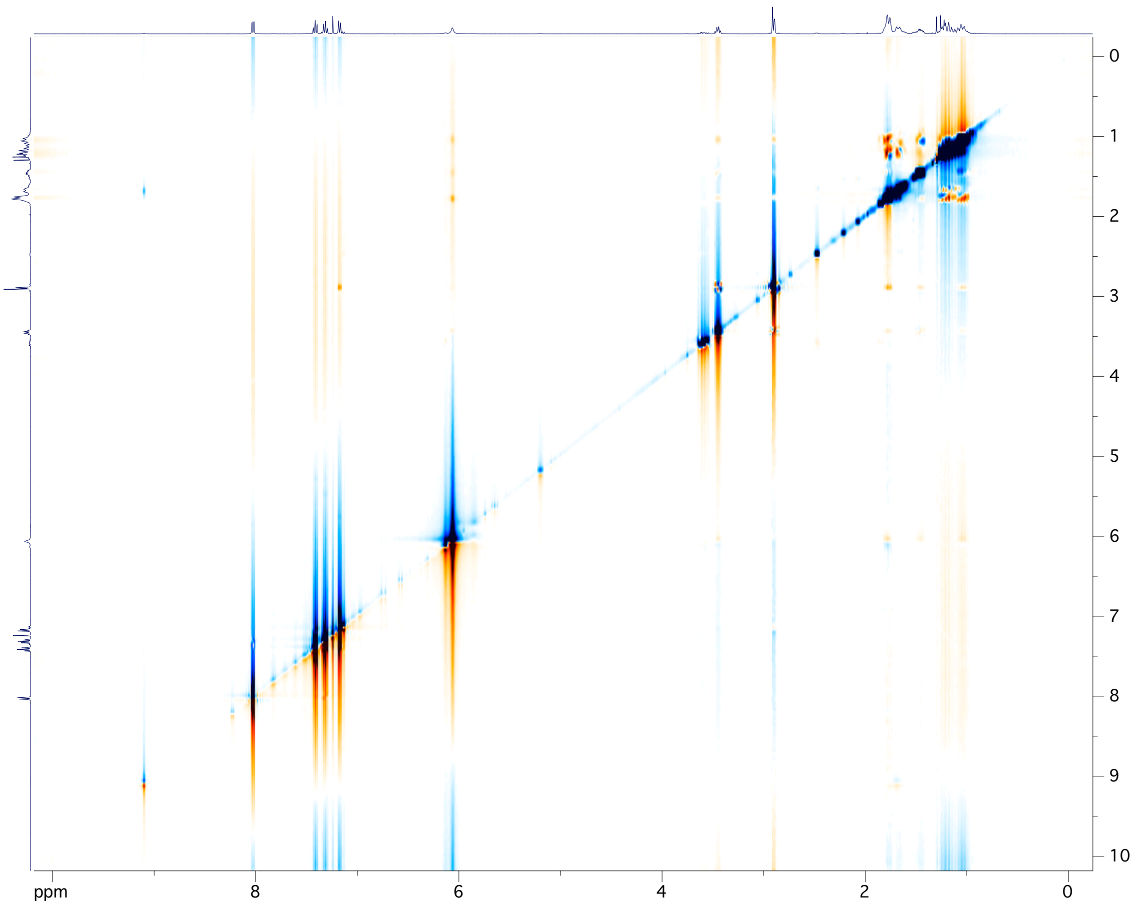

ppm
8
6
4
2
0
1
2
3
4
5
6
7
8
9
10

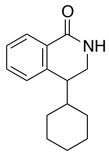
O
NH

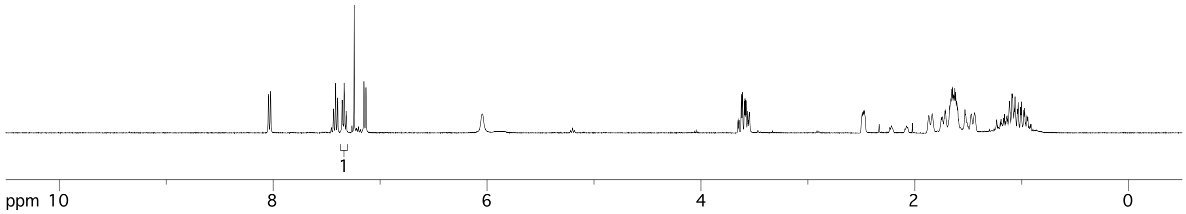
1
ppm 10
8
6
4
2
0

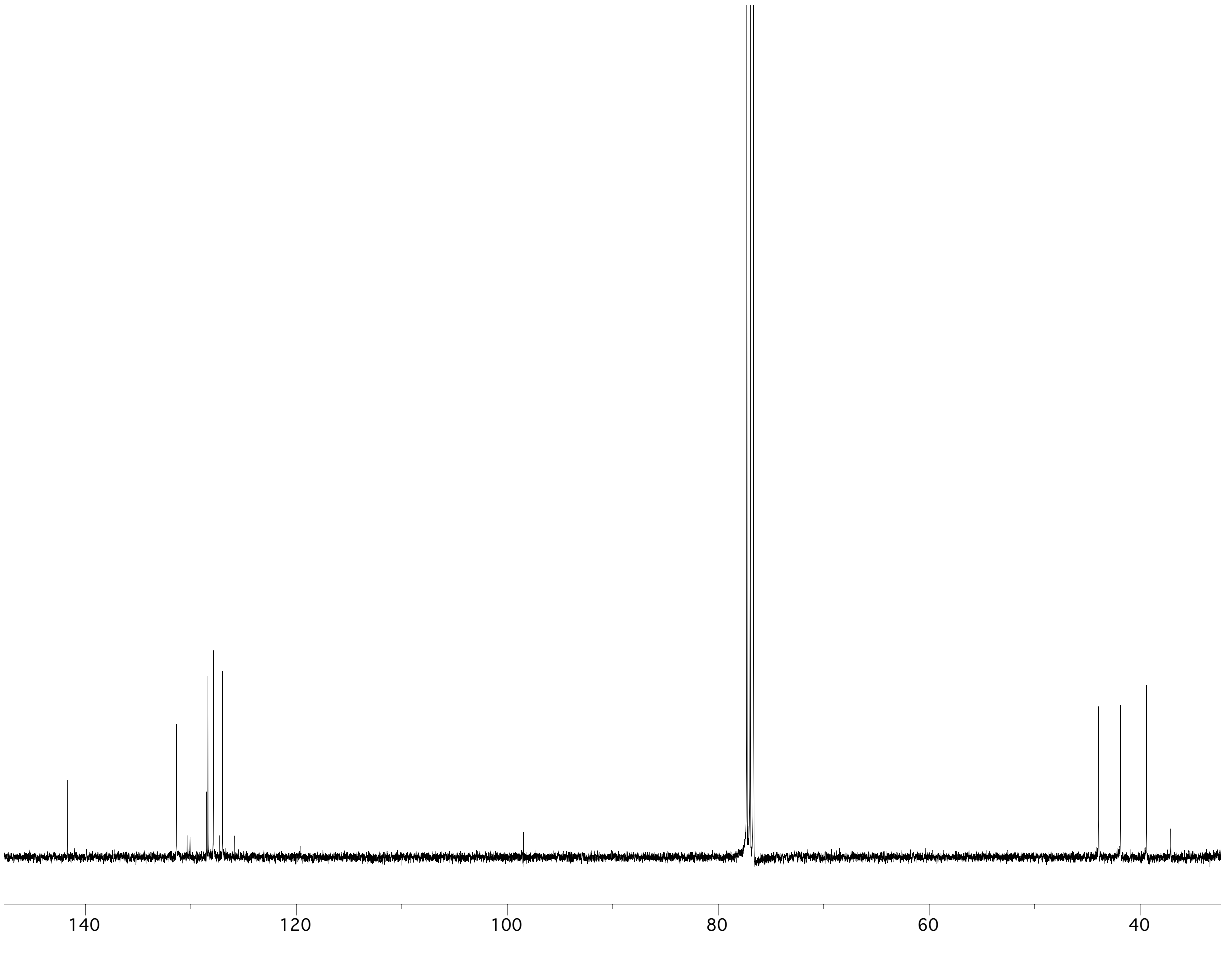
140
120
100
80
60
40

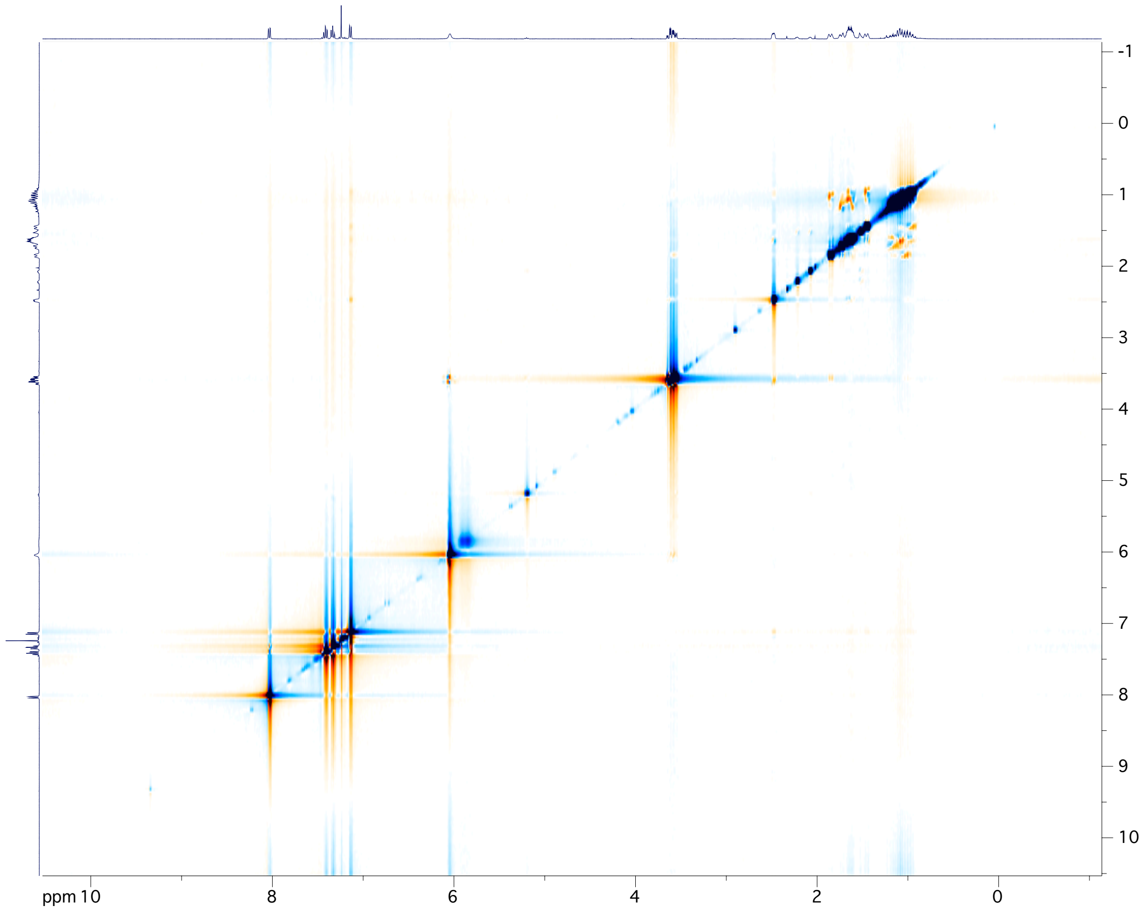
ppm 10
8
6
4
2
0
-1
0
1
2
3
4
5
6
7
8
9
10

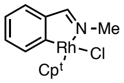
N
-Me
Rh
Cl
Cpt

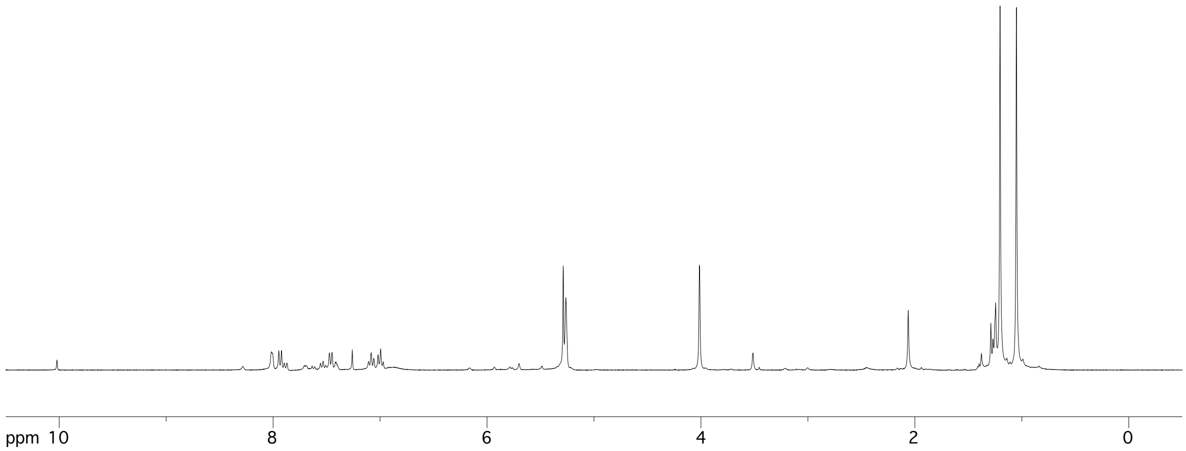
ppm 10
8
6
4
2
0
